# Synthesis Route to Single-Walled Zeolite Nanotubes Enabled by Tetrabutylammonium Hydroxide

**DOI:** 10.1021/acsmaterialsau.4c00030

**Published:** 2024-07-11

**Authors:** Anthony Vallace, Dhrumil R. Shah, Enerelt Burentugs, Atticus J. Tucker, Ashley E. Cavanagh, Christopher W. Jones

**Affiliations:** School of Chemical & Biomolecular Engineering, Georgia Institute of Technology, 311 Ferst Drive, Atlanta, Georgia 30332, United States

**Keywords:** zeolite nanotube, synthesis, isomorphous substitution, CO_2_ conversion, aromatics

## Abstract

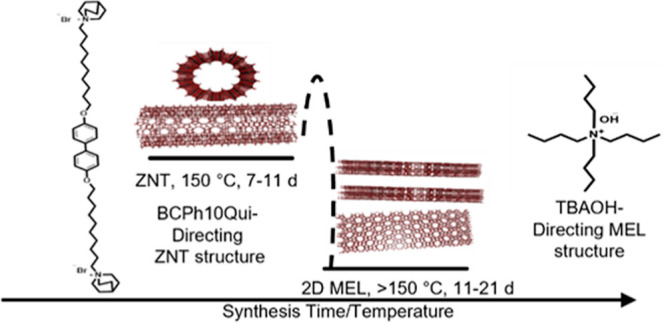

Single-walled zeolite nanotubes (ZNT) were recently synthesized in a narrow compositional window. ZNT structural features—thin zeolitic walls and large mesopores—can allow for easy access of small molecules to zeolite micropores, but they also impart processing limitations for these materials, such as challenges with conventional aqueous ion-exchange conditions. Conventional solid- and liquid-phase ion exchange of calcined NaOH-derived ZNT (NaH-ZNT) results in structural degradation to either 2D sheet-like phases, 3D nanocrystals, or amorphous phases, motivating different direct synthesis routes and unconventional ion-exchange procedures of uncalcined ZNT precursors. Here, a modified synthesis route for ZNT synthesis is introduced that facilitates facile ion exchange as well as incorporation of additional non-Al heteroatoms in the zeolite framework. Tetrabutylammonium hydroxide (TBAOH) is used as a hydroxide source and co-OSDA, enabling synthesis of new compositions of ZNT, otherwise unachievable by post-modification of previously reported NaH-ZNT. By varying the gel composition, synthesis temperature, crystallization time, hydroxide source, silicon source, and aluminum source, productive conditions for the new TBAOH synthesis are developed, leading to increased strong acid site density in the ZNT. The collected results demonstrate the sensitivity of the ZNT synthesis to many key parameters and show that the ZNT forms only when Si/(Al + T) ∼ 30 in these synthesis gels and with specific Si and Al sources, and always in the presence of trace Na^+^. Catalytic testing, via the tandem CO_2_ hydrogenation to methanol and methanol to aromatics reaction, shows that ZNTs provide adequate catalytic activity (acidity), relative to their conventional 3D counterparts in converting methanol to aromatic compounds.

## Introduction

Crystalline aluminosilicate zeolites are well-known microporous materials in catalysis, separations, ion exchange, and numerous other applications. Typically, zeolites exist as three-dimensional crystallites where the average diffusion length into the pores is on the micron scale, on the order of the size of the particle. The size of the crystallite domains can be reduced during zeolite synthesis by extended periods of low-temperature aging and lower crystallization temperatures for shorter crystallization times.^1^ To combat diffusion limitations, zeolite nanocrystals have been synthesized with long aging periods and the addition of small organic molecules or larger surfactant molecules, which are used to promote nucleation and limit crystal growth. For example, Debost synthesized CHA nanocrystals using a ternary mixture of Na^+^, K^+^, and Cs^+^, where the gel was aged at room temperature for 17 d, followed by crystallization at 90 °C for 8 h.^2^ Holmberg synthesized FAU nanocrystals using TMAOH and TMABr to study the effects of structure directing agent (SDA) counterion on the crystal size and yield. They showed that gels containing Br^–^ were 45% smaller by volume and obtained 73% more yield than the Br^–^—free gel, after 54 h of hydrothermal synthesis.^3^ Wang synthesized nanozeolite IM-5 1,5-bis(*N*-methylpyrrolidinium)pentane bromide as structure-directing-agent and poly(ethylene glycol) (PEG) and cetyltrimethylammonium bromide (CTAB) as additives and surfactants, respectively.^4^ Mintova has also demonstrated preparation of pure silica MEL nanocrystals using tetrabutylammonium hydroxide (TBAOH) as an OSDA in an ethanol/water cosolvent approach, where the addition of the cosolvent changed the solubility behavior of the silica precursors; different aging times on an orbital shaker were shown to influence the nanozeolite crystal size.^5^

Beyond zeolite nanocrystals are 2D ultrathin zeolites where the effective domain size is 1–2 unit cells thick, resulting in sheet-like structures.^6−9^ These materials can be further delaminated or exfoliated to give different stacking arrangements for these zeolite nanosheets. Corma synthesized delaminated ITQ-6 and Ti-ITQ-6 by first making FER in F^–^ media with 4-amino-2,2,6,6-tetramethylpiperidine as an OSDA. Once the product, “PREFER,” was obtained, it was subjected to exfoliation and delamination by mixing the uncalcined FER with CTAB and TPAOH in water with inclusion of ultrasonic treatment.^10^ The increased BET surface area and mesopore volume of ITQ-6 compared to 3D calcined “PREFER” were consistent with delamination and exfoliation. Similarly, Corma has demonstrated pillared structures of MCM-22, where a precursor zeolite is swollen with CTMA^+^, then treated with TEOS, which form SiO_2_ pillars after calcination.^6^

Two relatively recent hierarchical zeolite structures have been developed by the Rimer Group. Core-shell and egg-shell structures of MFI and MEL were prepared, where the core-–shell materials consisted of the pure silica analogue (silicalite-1 or silicalite-2) as an exterior shell and the aluminosilicate form (ZSM-5 or ZSM-11) as the core. Conversely, the egg-shell structures were the reverse with an aluminosilicate exterior and a pure silica interior. The mesoscopic gradients in core-shell materials resulted in faster diffusion to the inner acid sites than the homogeneous counterparts when used in hydrocarbon upgrading. The egg-shell materials existed as pseudo-nanosheets and gave higher turnover rates than the homogeneous counterparts.^11,12^ The Rimer group has also recently demonstrated the synthesis of finned zeolites, where fin-like protrusions are grown on a seed crystal with identical framework topology or can be synthesized directly. The finned zeolites (MFI and MEL) showed better catalytic performance in methanol to hydrocarbon catalysis than their conventional counterparts. The effective diffusive length was shown to be the distance between fins rather than the bulk particle size. The finned zeolites were synthesized directly by altering the water content of conventional MFI or MEL gels. Alternatively, finned zeolites were also prepared by first preparing a conventional MFI or MEL, then adding a secondary growth solution to the seeds prior to calcination.^13^

Another class of 2D zeolites are zeolite nanosheets, first reported by Choi, where the OSDA was designed as a diquaternary ammonium-type surfactant, C_22_H_45_–N^+^ (CH_3_)_2_–C_6_H_12_– N^+^(CH_3_)_2_–C_6_H_13_. With this surfactant, the ultrathin zeolite structure is said to form at the hydrophilic end and the hydrophobic tail restricts excessive zeolite growth. Furthermore, when zeolite synthesis is performed with the bromide form of the SDA and NaOH, a multilamellar mesostructure was produced, where the sheets had long-range order along the *b*-axis. In contrast, when the SDA was ion exchanged to the hydroxide form and Na^+^ was removed from the gel, a unilamellar morphology was obtained, where the sheet stacking was random and long-range order along the *b* axis was lost.^14^

Recently, a 1D form of zeolites was synthesized in the form of a single-walled zeolite nanotube (ZNT). First reported by Korde et al.,^15^ this synthesis used a unique di-quaternary ammonium surfactant (([1,1′-biphenyl]-4,4′-diylbis(oxy))bis(decane-10,1-diyl))bis(quinuclidin-1-ium) bromide (BcPH10Qui), somewhat similar to the molecule described above, with NaOH as the hydroxide source and Ludox SM-30 and Al_2_(SO_4_)_3_·18H_2_O as the SiO_2_ and Al_2_O_3_ sources. ZNT synthesis was performed at 150 °C for 7 days, and the resulting nanotube material has a Si/Al of ∼15, consisting of a unit cell thick BEA* outer wall and MFI inner wall, where the inner diameter of the tube is ∼3 nm. Like the 2D MFI materials developed by the Ryoo group,^14^ the SDA creates micelles due to the hydrophilic quinuclidinium head groups and the long hydrophobic alkyl chains. The two arene groups in the center of this symmetrical OSDA molecule are thought to π stack, making micellar structures that template the nanotube, on the mesoscale. This π stacking was supported by a shift and split in the UV–vis absorption spectra of solid OSDA and uncalcined ZNT compared to those of a dilute solution of OSDA. In addition to being a hydrophilic head, the quinuclidinium groups are also templating the zeolite phase. ICP OES of the calcined ZNT showed a Na/Al ratio of ∼0.6 and a bulk Si/Al ratio of ∼15.

ZNTs are structurally interesting and potentially useful 1D zeolites, but their thin walls make them labile in aqueous media after the SDA has been removed. Additionally, to date, they have been reported only as aluminosilicates at a fixed Si/Al ratio using the synthesis described above. The work reported here demonstrates new synthesis and ion exchange procedures that enable the synthesis of new compositions of ZNTs. The parameter space leading to a successful ZNT synthesis is also explored.

## Materials and Methods

### Synthesis of Structure Directing Agent (SDA) 1,1′-(([1,1′-Biphenyl]-4,4′-diylbis(oxy))bis(decane-10,1-diyl))bis(quinuclidin-1-ium) Bromide(BCPh10Qui)

The SDA BCPH10Qui synthesis was carried out by following our recently published procedures.^15^ Briefly, 1.6 g of 4,4′-biphenol, 12.5 g of 1,10-dibromodecane, 1.6 g of potassium hydroxide, and 25 mL of ethanol (200 proof) were refluxed overnight in a round-bottom flask, under Ar. Once complete, the reaction mixture was cooled to room temperature, and the resulting light blue solid was washed with excess hot (343–348 K) ethanol/water (50:50 v/v) solution to obtain the intermediate BCPH10Br. The intermediate product was then dried overnight on a high vacuum line. Next, 0.5 g of BCPH10Br, 0.35 g of quinuclidine, and 25 mL of dry acetonitrile was added to a round-bottom flask, with stirring, and was refluxed overnight under Ar. The reaction mixture was cooled to room temperature and poured into a large excess of diethyl ether to precipitate the BCPH10Qui product. The product was then washed with diethyl ether, isolated with vacuum filtration, and dried overnight at 303–308 K, under a vacuum.

### Synthesis of Zeolite Nanotubes

#### NaH ZNT

Zeolite nanotube synthesis, using NaOH as a mineralizer, was performed using our recently published procedure.^15^ First, 0.113 g of BCPH10Qui and 4.45 g of deionized water was added to a 30 mL polypropylene bottle and was stirred to obtain a homogeneous suspension. Next, 0.067 g of NaOH and 0.027 g of aluminum sulfate hydrate (Al_2_(SO_4_)_3_·14–18H_2_O) was dissolved in the reaction mixture. Lastly, 0.5 g of Ludox SM-30 colloidal SiO_2_ was added, giving a final gel composition of 18.75SiO_2_: 1BCPH10Qui: 0.3Al_2_O_3_: 6.3Na_2_O: 2050H_2_O. The gel was aged at room temperature with stirring for 3 h and then was transferred to a Teflon-lined autoclave and allowed to crystallize for 7 days at 423 K. The resulting solid separated with centrifugation, washed three times with deionized water, and dried in an oven at 348 K overnight. The solid was then calcined in stagnant air at 823 K (2 K/min) for 6 h. Sample notation for the NaOH mediated synthesis is NaH-ZNT, which refers to the calcined NaOH-derived zeolite nanotube, which was previously shown to be 60% Na^+^ 40% H^+^ (mol %) as Al balancing cations.

#### TBA-Al/T-ZNT-*x*

ZNT using TBAOH as a mineralizer were synthesized with the following gel compositions: 18.75SiO_2_: 1BCPH10Qui: 0.15–0.3Al_2_O_3_: 0.03–0.15T_2_O_3_: 9.5TBA_2_O: 2050H_2_O, where T = Fe^3+^ or B^3+^. Synthesis was performed as follows: 0.113 g of BCPH10Qui, TBAOH (Macron Chemicals 40% in H_2_O-1.65 g or Millipore Sigma 20% in H_2_O-3.3 g), 0.0135–0.027 g of Al_2_(SO_4_)_3_·14–18H_2_O, 0.0019–0.0086 g of Fe_2_(SO_4_)_3_·H_2_O, 0.25 g of boric acid solution (1 wt % in H_2_O), and 1.5–3.1 g of DI H_2_O, (18.2 MΩ) was added to a 30-mL polypropylene bottle under stirring to homogenize. Lastly, 0.5 g of Ludox SM-30 was added to the mixture, which was allowed to age for 3 h at room temperature then transferred to a 38 mL Anton Parr acid digestion bomb and crystallized for 7 to 21 days (9 to 11 days is optimal) at 423 K. The resulting solid was separated by centrifugation, washed three times with deionized water, and dried in an oven at 348 K overnight. The solid was then calcined in stagnant air at 823 K (2 K/min) for 6 h. The sample notation for TBAOH mediated synthesis is TBA Al ZNT (Al only synthesis) or TBA Al/T ZNT-*x*, where T = Fe or B and *x* is the molar ratio of Al to T in the gel. A full summary of the sample nomenclature is shown in Table S1. The synthesis was successfully scaled up three times.

#### Ion Exchange

All ion exchange experiments were performed on NaH ZNT. Initially conventional zeolite ion exchange procedures were performed on calcined NaH ZNT to obtain an H ZNT using 1 M NH_4_NO_3_ at 353 K for 3 h three times. The NH_4_NO_3_ concentration was also reduced to 0.1 and 0.01 M at 353 K for 3 h, three times (100 mg/50 mL solution). HCl exchange was performed using 100 mg of ZNT and 5 cc of 0.02 M HCl (10-fold molar excess) at 90 °C for 3 h. Solid-state ion exchange was performed with various chlorides on calcined NaH ZNT (LiCl, NaCl, NH_4_Cl, and CaCl_2_), where the salt and NaH ZNT were thoroughly mixed with a mortar and pestle, with a small amount of anhydrous ethanol added as a dispersant, The mixture was placed in a tube furnace, heated to 423 K (2 K/min) for 2 h, and then raised to 823 K for 6 h. Modified ion exchange procedures were then adopted. Uncalcined NaSDA ZNT was contacted with 1 M NH_4_NO_3_ or 1 M NaNO_3_ at 353 K for 3 h three times, followed by calcination at 823 K for 6 h.

#### Preparation of BEA and MFI

The proton form of ZSM-5 (MFI) and BEA* were prepared from the ammonium cationic form provided from Zeolyst International Inc. (CBV2314 and CP814E). The zeolites were calcined in air at 550 °C (heating ramp rate of 2 °C/min) for 6 h.

### Characterization

#### XRD

Ex situ XRD was performed using a Rigaku Miniflex equipped with a Toshiba A-20 Cu X-ray tube using K-α radiation (λ = 1.5406 Å) and a HyPix-400 MF 2D hybrid pixel array detector (HPAD). Diffraction data were collected from 5–50° 2θ at 5°/min with a step-size of 0.01°. Typically, ∼15 mg of sample was mounted on a Si(510) low background sample cell, under ambient lab conditions, using a glass microscope slide. ZNT Bragg peaks were indexed using previously reported data.^15^

In situ XRD was conducted using a Rigaku Smartlab XE XRD equipped with a PhotonMax high-flux 9 kW rotating anode X-ray source (Cu k-α radiation, λ = 1.5406 Å), an in-plane arm (5-axis goniometer), a 2.5° solar slit, and a HyPix-3000 high energy resolution 2D HPAD detector. Typically, ∼15 mg of sample was mounted on a quartz sample cell and was loaded into a Reactor *X* high temperature attachment for reactive gases. Data was collected under N_2_ flow (70 SCCM, LN_2_ source) using the following procedure: the cell was purged with N_2_ for 60 min and an initial scan (5–50° 2θ at 5°/min, step-size-0.01°) was taken at 25 °C then the temperature was ramped to 150, 200, 300, 400, 500, 600, 700, and 800 °C at 10 °C/min. Each temperature was held for 30 min before a scan was taken. For humid experiments, the N_2_ stream was humidified with a Licor Li-610 humidifier, and the same ramping procedure was conducted.

#### NH_3_ TPD

Ammonia temperature-programmed desorption (NH_3_ TPD) was performed using a Micromeritics Autochem 2920 instrument. First, the samples were preheated to 673 K under helium to remove water and other volatile species. The samples were then flushed with ammonia at 313 K for 60 min, followed by removal of the physiosorbed species under helium for 1 h. The sample was heated under helium at 10 to 673 K/min, and the desorbed species were recorded using a TCD detector.

#### IPA TPD

Isopropyl amine temperature-programmed desorption (IPA TPD), first reported by Gorte,^16^ was used to quantify the Brønsted acid sites (BAS) concentration. IPA selectively reacts over BAS to generate propylene and ammonia, whereas Lewis acid sites (LAS) are inactive, desorbing unreacted IPA. Experiments were performed by using an in-house fixed-bed setup connected to a mass-spectrometer (Pfeifer Vacuum GSD-320) to measure real-time gas concentrations. A known mass of zeolite (∼50–100 mg) was pelletized and loaded onto the fixed bed between a layer of SiC grit and quartz wool. The zeolite was then activated under flowing N_2_ at 400 °C for 1 h (5 °C/min). The sample was then cooled to 100 °C (5 °C/min). Isopropyl amine was then added with 3 × 50 μL injections using a N_2_ carrier gas. Once sufficient time was given for the removal of the nonadsorbed and weakly adsorbed IPA (as confirmed by the absence of IPA signals on the mass-spectrometer), the sample was heated at a constant rate of 10 °C/min to 700 °C under flowing N_2_ (30–50 SCCM). The signals corresponding to propylene (*m*/*z* = 41), isopropyl amine (*m*/*z* = 42), and ammonia (*m*/*z* = 17) were tracked in real-time with the Faraday sensor on the online mass spectrometer connected to the outlet of the fixed bed. Using the ionic displacement versus time curve, a concentration versus time curve was made for propylene and the overall moles, and hence, the concentration of BAS on the catalyst sample was calculated by integrating the propylene signal.

#### ICP-OES

Elemental analysis was performed by Galbraith Laboratories using inductively coupled plasma optical emission spectroscopy (ICP-OES). The following compositions were determined: The Si/Al ratio, Na/Al ratio, and Al/T ratio (T = Fe^3+^ or B^3+^).

#### Catalytic Testing

The zeolites were tested for the combined CO_2_ hydrogenation/methanol aromatization activity in tandem with ZnO–ZrO_2_.^17,18^ ZnO–ZrO_2_ (synthesized using coprecipitation method) and zeolite powders were mixed and pelletized in 1:2 w/w ratio. The pelletized sample was then packed in a 1/4″ SS316 tube in between a SiC bed (XRD of pelletized ZNT Figure S10). A CO_2_, H_2_, and N_2_ gas mix in the ratio (11:33:56, Matheson) was passed over the catalytic bed and maintained at a pressure of 600 psi using a Tescom ER3000 backpressure regulator. An in-line Agilent 7890 gas chromatograph was used to analyze the product distribution after CO_2_ hydrogenation and methanol conversion to hydrocarbons. Data were collected at steady state after 10 h of run time.

#### N_2_ Physisorption

The porosity properties of the ZNT samples were measured by using a Micromeritics Tristar II plus. Samples were degassed at 423 K in a homemade degas oven connected to a vacuum manifold (10^–3^ Torr). The N_2_ isotherms were collected at 77 K and the BET surface areas were determined using the gas uptake from 0.05 to 0.3 *P*/*P*_0_. The total pore volume of ZNT, which was used to assess degrees of degradation and nanotube phase purity, was determined by converting the volumetric N_2_ uptake at 0.99 P/P_0_ to the equivalent volume of liquid nitrogen using a density of 0.807 g/cm^3^. Note: select samples were also run on a Micromeritics ASAP 2020, where they were degassed at 423 K, under high vacuum (10^–6^ Torr). No discernible difference between the ASAP isotherms and Tristar isotherms was observed, suggesting the rough vacuum degas system, used for the Tristar, was sufficient activation conditions to give reliable micropore information about the zeolite samples.

#### Diffuse Reflectance Ultraviolet–Visible (DRUV–Vis) Spectroscopy

DRUV–vis spectroscopy was used to qualitatively determine the types of Fe species present in TBA-Al/Fe-ZNT-*x*. Spectra were recorded under ambient conditions, using Cary 5000 UV/vis NIR spectrometer. Powder samples were packed in the sampler and studied in the range of 200–800 nm.

#### Nuclear Magnetic Resonance

All solid-state nuclear magnetic resonance (ssNMR) experiments involved packing ∼50 mg of sample into a 4 mm ZrO_2_ NMR rotor and spinning at 10 kHz.

^29^Si MAS NMR was performed by using a Bruker AVIII-HD 300 MHz solid-state spectrometer. The dwell time was set at 16 μs with a pulse delay of 2 s. Each ssNMR run was measured for 1024 scans. The peaks were deconvoluted and integrated in TopSpin allowing for calculation of the Si/Al ratio using the following equation.
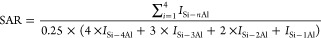


^1^H MAS NMR was performed by using a Bruker AVIII-HD 300 MHz solid-state spectrometer and was collected prior to the ^29^Si MAS spectrum. ^1^H MAS NMR used a dwell time of 1 μs with a pulse delay of 1 s; four scans were taken for each ^1^H MAS spectrum.^27^Al MAS NMR was performed using a Bruker Avance III 400 MHz spectrometer with a dwell time of 1 μs, a pulse delay of 1 s, and 8192 scans. Samples were hydrated after packing in the rotor using a micropipet.

#### Transmission Electron Microscopy

Transmission electron microscopy (TEM) along with energy-dispersive X-ray spectroscopy were performed using a Hitachi HD2700 with a 200 kV accelerating voltage and a spherical aberration (Cs) corrected cold field emission source. Samples were prepared by using carbon-coated copper grids using an ethanol suspension.

## Results and Discussion

### Stability Experiments

Early in the work with ZNTs, it was observed that conventional solid and liquid phase ion exchanges on calcined NaH ZNTs resulted in framework degradation. To determine the cause of degradation, NaH ZNT samples were subjected to treatment in boiling water, acetonitrile, or hexane (polar-protic, polar-aprotic, nonpolar) for 18 h. Treatment in boiling water resulted in a 38% loss in pore volume, suggesting that the ZNT was degrading to lower energy and denser phases. A reduction pore volume suggests framework degradation (Figure S1). Degraded material can also be seen in the TEM (Figure S1, circled in red), and the hot liquid water (HLW) treated ZNTs appear shorter in length compared to neat NaH-ZNT. Treatment in acetonitrile and hexane resulted in less degradation, where a 21 and 24% reduction in pore volume was observed, respectively. HLW treatment is known to degrade BEA*, especially when the zeolite contains high defect concentrations (>400 μmol/g) or a high Al content (SAR ∼ 14–19), both of which apply to ZNT.^19−21^ In the case of HCl ion exchange, there is also likely some desilication occurring, contributing to the degradation, as seen in the ^29^Si MAS NMR spectra (Figure S2) of NaH-ZNT before and after HCl treatment, where the calculated SAR reduces from 15 to 8. The structure of the ZNT is such that intraparticle diffusion limitations are presumably nonexistent, causing T atom removal to occur much faster than that with conventional zeolites under the various liquid treatment conditions. In addition, because the ZNT walls are only about one unit cell thick, T atom removal is likely to result in structural collapse rather than local point defects that would occur in conventional 3D zeolites. TEM images of degraded nanotubes are shown in Figures S1. The ZNTs were conversely quite stable in the gas phase under high temperatures, as seen in the in situ XRD patterns up to 800 °C under dry or humid N_2_ (Figure S3).

### Ion Exchange Studies

Unconventional ion-exchange procedures were thus developed, where the uncalcined nanotubes (Na-SDA-ZNT) were ion exchanged with NH_4_^+^ (NaH-ZNT-NH_4_^+^-ex) or TBA-SDA-ZNT was ion exchanged with Na^+^ (TBA-Al-ZNT-Na^+^-ex) followed by calcination. Our previous work showed that NaH-ZNT contains 60 mol % Na^+^ and 40 mol % H^+^ cations (BAS), charge balancing cations, where the BAS arise from decomposition of the charge balancing quinuclidinium groups in the NaSDA-ZNT. The H^+^/Al ratio of 0.4 and SAR of 15 of NaH ZNT, equates to ∼17 wt % of BCPh10Qui that is balancing framework charge in the NaSDA ZNT. TGA combustion experiments show a ∼ 43 wt % loss of SDA upon calcination (Figure S4). The remaining 26 wt % SDA that is not balancing charge is assumed to be either occluded inside the mesopore of the nanotube, confined by van der Waals forces, or located in the internanotube volume. This means that some of the large BCPh10Qui surfactant molecule cannot be exchanged with Na^+^ or NH_4_^+^ due to steric constraints. Similar steric constraints were observed by Choi^14^ when attempting to extract a similar surfactant SDA from MFI nanosheets using HCl. Because ion exchange must be performed on uncalcined ZNT to retain the nanotube structure, the maximum achievable exchange level is limited by the amount of quinuclidinium groups balancing framework Al (∼40 mol %) using that procedure.

### Direct Synthesis of Low Na^+^, H^+^-Rich ZNT

Sodium plays a role in both the atomic arrangement of Al T atoms and the mesoscale arrangement of nano-domains. For example, the effect of Na^+^ in synthesis gels on the Al arrangement of CHA, MFI, and MEL was described in a series of papers from Gounder and coworkers. Their findings for all three frameworks demonstrated that Al–Al pairs tended to form when a single monovalent cation was occupying a certain extra framework cation position, whereas site-isolated Al sites often formed when an organic and inorganic cation competed for the same extra framework cation position. For MFI and MEL, this means that Al–Al pairs tend to form in the organic-rich (low Na^+^) gels by using TPAOH and TBAOH, respectively. Conversely, single-site Al formed in CHA (SSZ-13) in the organic-rich gels using TMAda^+^—rich gels.^22−25^Table 1 shows a summary of the effect of Na^+^ on the resulting zeolite product during the ZNT synthesis. ZNT was obtained using the originally reported NaOH route^15^ and the new TBAOH route described here. When TBAOH is used, the total base/SiO_2_ ratio is increased from 0.4 (NaOH route) to 0.6 due to the weaker basicity of TBAOH (*K*_b_ = 0.27) compared to NaOH (*K*_b_ = 0.63).

**Table 1:** Summary of the Effect of Na^+^ on ZNT Synthesis

sample description	crystallization time (days)	SiO_2_ source	Na^+^:TBA^+^	Base/SiO_2_	result
NaH ZNT	7	Ludox SM-30	Inf	0.4	NaHZNT
larger particle colloidal SiO_2_	7	Ludox HS-30	Inf	0.4	amorphous
TEOS Na ZNT	7	TEOS	Inf	0.4	amorphous
TBA-TEOS-ZNT	11	TEOS	0	0.6	amorphous
TBA-Al ZNT	11	Ludox SM-30	0.04^a^	0.6	ZNT
TBA, TEOS trace NaOH	7	TEOS	0.04	0.6	ZNT^b^
TBA, TEOS Trace NaCl	7	TEOS	0.04	0.6	ZNT^b^
TBA, NaOH co-SDA	11	Ludox SM-30	0.09	0.6	ZNT^b^
	11	Ludox SM-30	0.15	0.6	ZNT
	11	Ludox SM-30	0.31	0.5	ZNT^b^
	11	Ludox SM-30	0.58	0.4	ZNT
	11	Ludox SM-30	1.7	0.4	ZNT
	7	Ludox SM-30	2.2	0.4	ZNT
	7	Ludox SM-30	5.2	0.4	ZNT

a Na^+^ is present in Ludox, additional Na^+^ not added.

b Incomplete crystallization [based on XRD and N_2_ physisorption (Figure S5)].

**Figure 1:**
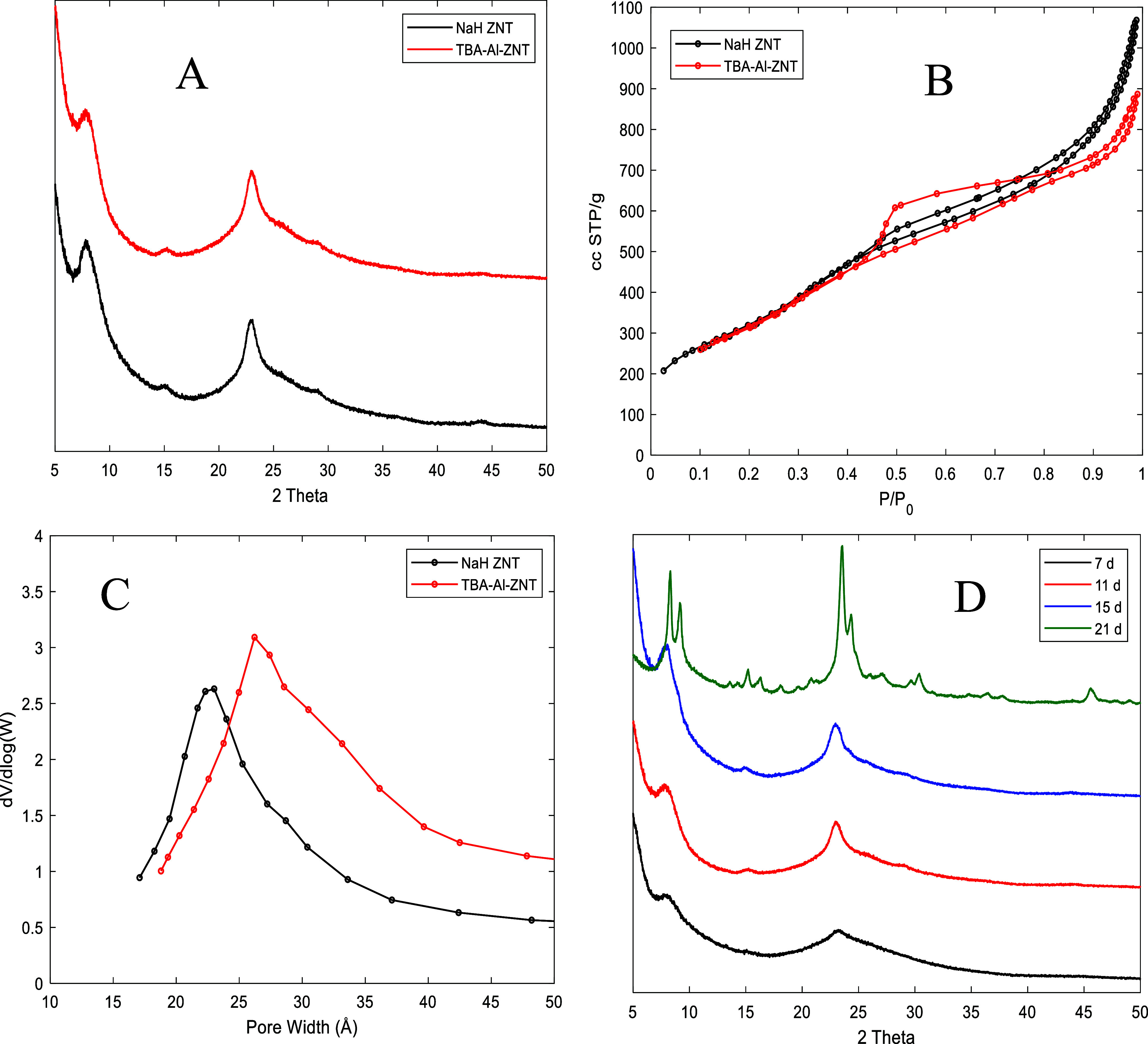
(A) XRD patterns of NaH ZNT and TBA–Al ZNT after 7 and 11 d crystallization time, respectively (B) N_2_ physisorption isotherms, (C) BJH pore size distributions of NaH ZNT and TBA–Al–ZNT, and (D) XRD patterns of TBA Al–ZNT at different crystallization times.

Because the total base/SiO_2_ ratio (0.4 and 0.6) and crystallization time (7 and 11 d) are different for the NaOH and TBAOH routes, the [SDA + TBA_2_O + Na_2_O]/SiO_2_ ratio also varied for the mixed NaOH/TBAOH samples (more TBA-rich = more total base). Diffraction patterns for the mixed TBA/Na ZNT series were similar and the determination of the presence or absence of amorphous phases (i.e., incomplete conversion) was inconclusive using XRD. Instead, a reduction in total pore volume suggested that the some of the samples were not completely crystallized in 11 d at 150 °C. Samples with less total base (more Na^+^-rich) showed N_2_ isotherms similar to NaH–ZNT, suggesting the total base content was not the only factor in the crystallization rate and a more complex phenomena could be occurring, where TBA^+^ and Na^+^ could be competing for cation sites, slowing crystallization in the more TBA^+^-rich systems. Interestingly Na/TBA-Al-ZNT-0.09, which had a base/SiO_2_ of 0.6, only reached ∼65% crystallinity in 11 days, suggesting co-occlusion, apparently slowing crystallization. The total base content was a factor in Na/TBA–Al–ZNT-0.31, as this sample was ∼85% crystallized as compared to Na/TBA–Al–ZNT–0.15, where the total base/SiO_2_ ratio was 0.5 and 0.6, respectively. Figure 1 shows the XRD and N_2_ physisorption data for NaH ZNT and TBA–Al ZNT.

**Figure 2:**
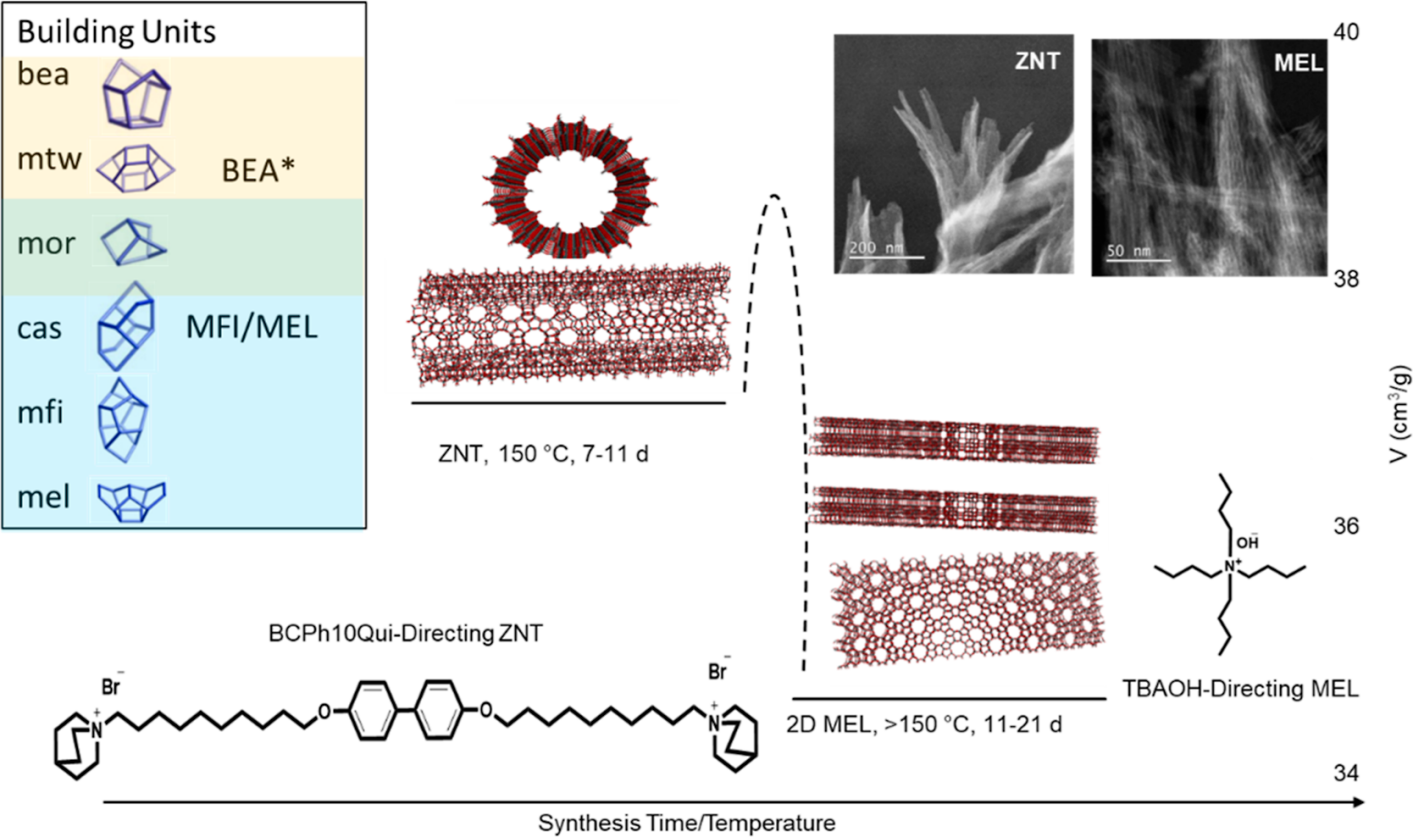
Stages of the TBA–BCPh10Qui-zeolite phase transition with increasing synthesis time, temperature, and pH, following paths of decreasing molar volume.

There is no discernible difference in the diffraction patterns between NaH-ZNT and TBA-Al-ZNT, though differences are observed in the N_2_ isotherms. The N_2_ uptakes at low *P*/*P*_0_ are identical, suggesting the same approximate purity of the zeolite phase; however, a difference in the mesopore uptake region is observed. NaH–ZNT shows a relatively small hysteresis loop and a total pore volume of 1.7 cc/g, whereas TBA-Al–ZNT shows a large hysteresis loop and a slightly lower total pore volume of 1.4 cc/g. Analysis of the BJH differential pore volume shows a small increase in the pore size distribution. Based on the observed porosity differences and results reported in the literature^14,26^ on mixed Na^+^ and organic cation systems, we propose that the NaH–ZNT consists of nanotubes arranged in an ordered multilamellar meso-structure and TBA–Al–ZNT is arranged in a more disordered unilamellar mesostructure. The disordered unilamellar arrangement is thermodynamically higher in energy than the multilamellar arrangement and the transition from unilamellar to multilamellar has been reported in MFI nanosheets.^26^ This transition is explained by an Oswalt ripening process, where the complete alignment of the surfactant molecules is thermodynamically preferred over a random orientation. Another contributor to lower total pore volume in TBA-Al-ZNT is the presence of extra-framework aluminum (EFAl) species (observed in ^27^Al MAS NMR, Figure 4C,D), which are not observed in NaH-ZNT.^15^ For TBA-Al-ZNT, when the crystallization time is extended past 11 days or the synthesis temperature is increased to 160 °C, ripening occurs directly to a 2D MEL phase, bypassing a multilamellar ZNT phase. A schematic of reaction coordinate versus framework density (expressed as molar volume) for the TBA^+^/BCPH10Qui system is shown in Figure 2.

Similar ripening was observed in Fe^3+^ containing TBA ZNT gels (discussed below, Figure S7). Co^2+^ titration is typically performed to assess the proximity of Al T sites as a function of the Na^+^/TBA^+^ ratio. This experiment cannot be reliably performed on ZNTs because exchange of calcined samples would degrade the nanotube, and complete Co^2+^ exchange of uncalcined ZNTs is currently not possible because of the inability to exchange/extract out the bulky BCPh10Qui surfactant.

The TBAOH synthesis is prone to densification to MEL if the oven temperature is raised to 160 °C or if the crystallization time is greater than 11 d. If larger ovens are used where temperature gradients can occur, the autoclave should be placed in the center to front of the oven to ensure that overheating does not occur when the oven temperature is set to 150 °C (assuming the heat source is in the back). At the gel composition of TBA_2_O/SDA = 9.5 and Si/(Al + T) = 30, crystallization time is fastest for B containing gels, then Fe-containing gels and slowest for Al-only gels, meaning that they can also densify to MEL at rates in this order. This is presumably due to Al_2_(SO_4_)_3_ being the strongest acid to neutralize some of the OH^–^, catalyzing the crystallization, followed by Fe_2_(SO_4_)_3_, and last B(OH)_3_. This difference in trivalent salt acidity can be accounted for by adjusting the water content at constant TBA_2_O/SDA; however, this was not performed in this work.

### Effect of T Atom Substitution

To assess the effect of different trivalent T atom substitutions, a series of TBA–ZNT samples was synthesized at constant Si/(Al + T)_gel_ and variable (Al/T)_gel_ (T = Fe^3+^ or B^3+^). A summary of different Fe and B containing ZNT samples is shown in Table 2 with their total acid site concentrations and measured BAS to strong acid site ratio.

**Table 2:** Summary of T Atom Substitution for ZNT Synthesized with Ludox SM-30, where Si/(Al + T)_gel_ = 30, Temperature = 150 °C, Crystallization Time = 7–11 d

sample description	Al/T (gel)	result^a^	MAS^c^ (μmol/g)	SAS^c^ (μmol/g)	calculated Si/(Al + T)^c^^,^^d^	BAS^e^:SAS^b^(%)
NaH-ZNT^15^	∞	ZNT	669	147	19, 15	100
TBA Al ZNT	∞	ZNT	501	176	23, 11	43
TBA Al/Fe ZNT-1	1	ZNT	291	141	37, 10	31
TBA Al/Fe ZNT-2	2	ZNT	489	257	21, 12	22
TBA Al/Fe ZNT-4	4	ZNT	625	218	18, 13	28
TBA Al/Fe ZNT-8	8	ZNT	841	104	16, 11	29
Na Al/Fe ZNT-1	1	ZNT^b^				
Na B ZNT	0	LEV^b^				
Na Al/B ZNT-1	1	LEV^b^				
TBA B ZNT	0	MEL				
TBA Al/B ZNT-1	1	ZNT	647	156	19, 12	34
TBA Al/B ZNT-2	2	ZNT	993	181	13, 10	37
TBA Al/B ZNT-4	4	ZNT	587	313	17, 10	21
TBA Al/B ZNT-8	8	ZNT	607	322	17, 11	19

a Confirmed with XRD and N_2_ physisorption.

b Significant amorphous phase (incomplete crystallization).

c NH_3_ TPD, includes strong and medium sites.

d Determined from ^29^Si MAS NMR.

e Determined from IPA TPD.

The optimal crystallization time for TBA–Al/Fe–ZNT was 9–11 d and TBA–Al/B–ZNT was 8–9 d. The rate of crystallization to ZNT and subsequent densification to MEL was fastest with B containing gels, Fe containing gels, and then Al-only gels, likely due to differences in pH, where the less acidic Fe_2_(SO_4_)_3_ or B(OH)_3_ precursors neutralize less OH^–^ at constant TBA_2_O/SDA, increasing the crystallization rate. Nanotubes were obtained using different Al/T ratios; however, it was observed that the Si/(Al + T) must be 30 to obtain high purity ZNTs. In NaOH gels, reduction in the silicon to aluminum ratio (atomic Si/Al, SAR) (gel) results in mostly amorphous phases. Al-free gels where Si/Fe = 30 also result in an amorphous phase. In borosilicate gels (Al-free), when Si/B = 30, LEV is obtained in the NaOH route, and MEL in the TBA route. Pure silica gels with TBA gave MEL and NaOH gave RUB.

**Figure 3:**
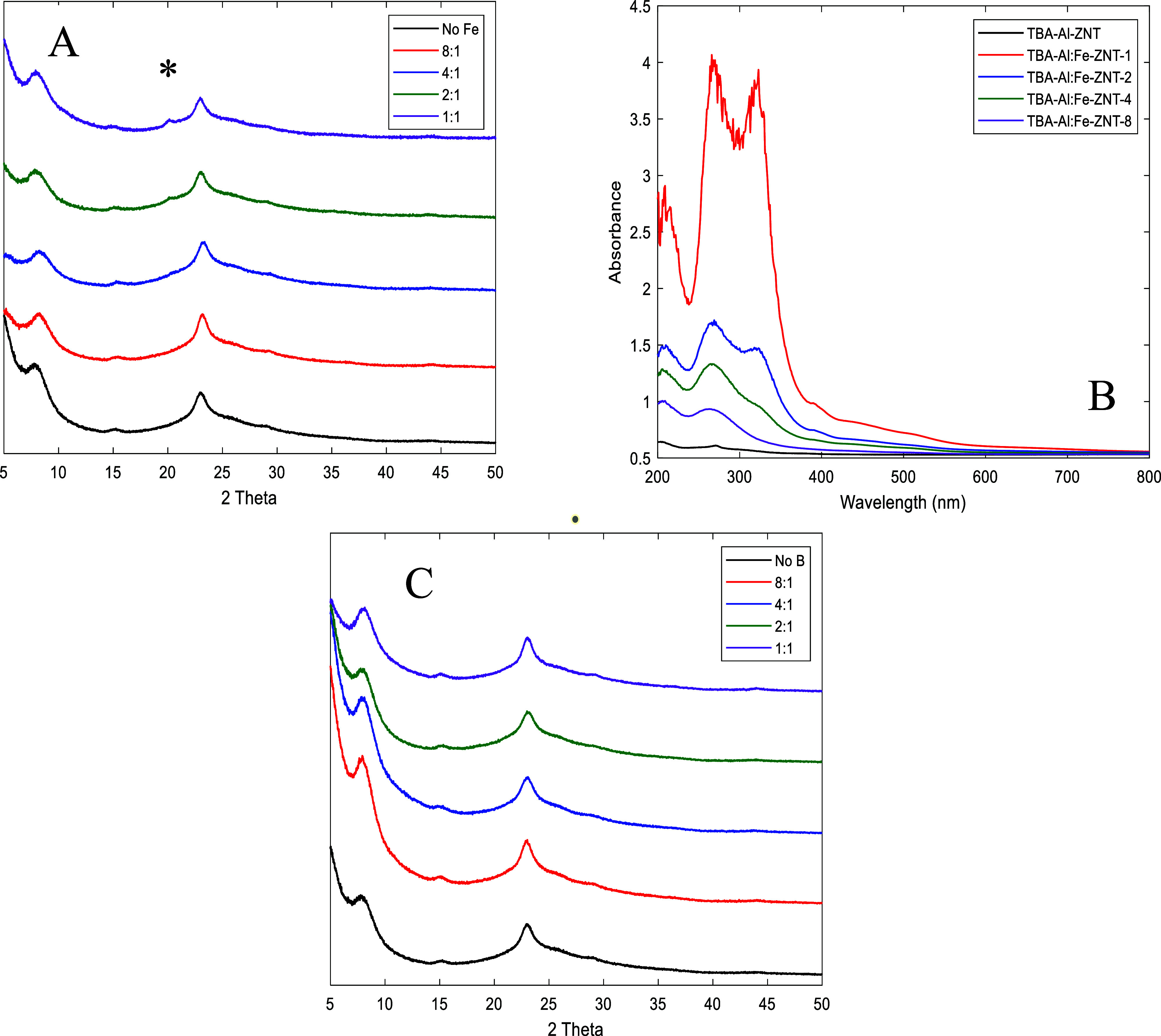
(A) XRD patterns and (B) DRUV–vis spectra of TBA–Al–ZNT and TBA–Al/Fe–ZNT 8–1 (C) XRD patterns of TBA–Al–ZNT and TBA–Al/B–ZNT-8 through1; *: Na_4_Al_3_FeO_8_.

XRD patterns for the TBA Al/Fe ZNT series (∞-1), shown in Figure 3A, all show the three Bragg peaks assigned to ZNT (8.1, 15.1, and 23.1° 2θ). In addition, TBA-Al/Fe-ZNT-1 and -2 show a Bragg peak at ∼20.1° 2θ. This peak is assigned to Na_4_Al_3_FeO_8_ [0 1 1], which is theoretically observed at 20.3° 2θ.^27^ The Na, which is present in the Ludox-SM-30 SiO_2_ source (SiO_2_:Na_2_O = 50), appears to form Na_4_Al_3_FeO_8_ as an extra-framework domain upon calcination at 550 °C. The Na/Fe ratio in the gels of TBA-Al/Fe-ZNT-1, -2, -4, and -8 were 2.4, 3.7, 6.3, and 11 respectively, implying that formation of the Na_4_Al_3_FeO_8_ is feasible from a stoichiometric perspective. Other crystalline Fe-oxide phases, such as Fe_2_O_3_ (α, β, γ, or ε), Fe_3_O_4_, or FeO were ruled out, as they do not contain a Bragg peak at ∼20.1° 2θ.^28,29^ TBA-Al/Fe-ZNT-8 and -4 both show a shift in the first Bragg peak (8.1° 2θ) to higher 2θ values, suggesting lower *d*-spacings, due to the isomorphous substitution of Fe^3+^ into the zeolite lattice. The trend in XRD peak shift is correlated with the appearance of the Na_4_Al_3_FeO_8_ peak, where lower Fe loadings (TBA-Al/Fe-ZNT-8 and -4) show the 2θ shift and no Na_4_Al_3_FeO_8_ peak, whereas the higher Fe loadings (TBA-Al/Fe ZNT- 2 and -1) do not show a shift in the ZNT Bragg peaks, but contain the Na_4_Al_3_FeO_8_ peak. These data suggest that use of excessive Fe^3+^ in the synthesis gel does not lead to significant lattice incorporation of Fe^3+^, and instead produces mostly non-silicate iron oxide phases.

NH_3_ TPD experiments on the ZNTs show three desorption peaks associated with strong, medium, and weak acid sites.^30,31^ Because NH_3_ nonselectively sorbs to many types of sites, these can consist of physisorbed or weakly chemisorbed NH_3_ (weak sites), BAS (strong, medium or weak sites), or LAS, which can consist of Na^+^ ions (weak sites) or EFAl species (strong or medium sites). Another possible Lewis acid site is zeolitic Al, Fe, or B, where the T(OH)Si bridge (BAS) has resonance structures consisting of Si–O^–^(H^+^)–T^–^ or SiOH → T (T = Al^3+^, Fe^3+^, or B^3+^), or dehydroxylated BAS.^32^ Weak acid sites may also be associated with external silanols,^32,33^ which are present in high concentration in ZNTs, due to the single unit cell thickness of the tube walls. The Si/(Al + T) ratios calculated from NH_3_ TPD (assuming 1 mol of H^+^/mol of T^3+^) were higher than those determined from NMR. This could be due to the exclusion of weak acid sites (∼90 °C desorption temperature), which are likely to include physisorbed NH_3_ and weakly chemisorbed NH_3_, in calculating the SAR. The difference in calculated SAR from NH_3_ TPD (higher values) and ^29^Si MAS NMR (lower values) suggests some acid sites are convoluted with physisorbed NH_3_. Interestingly, IPA TPD showed that ∼100% of the strong acid sites in NaH–ZNT were BAS; however, all TBA-derived ZNTs showed significant LAS. The LAS in TBA–Al–ZNT can best be assigned to EFAl. In the Fe-containing samples, EFAl, cationic Fe and extra framework Fe_*x*_O_*y*_ species are likely the LAS. In the B-containing samples LAS could be from both EFAl and extra framework B-oxides.

**Scheme 1:**
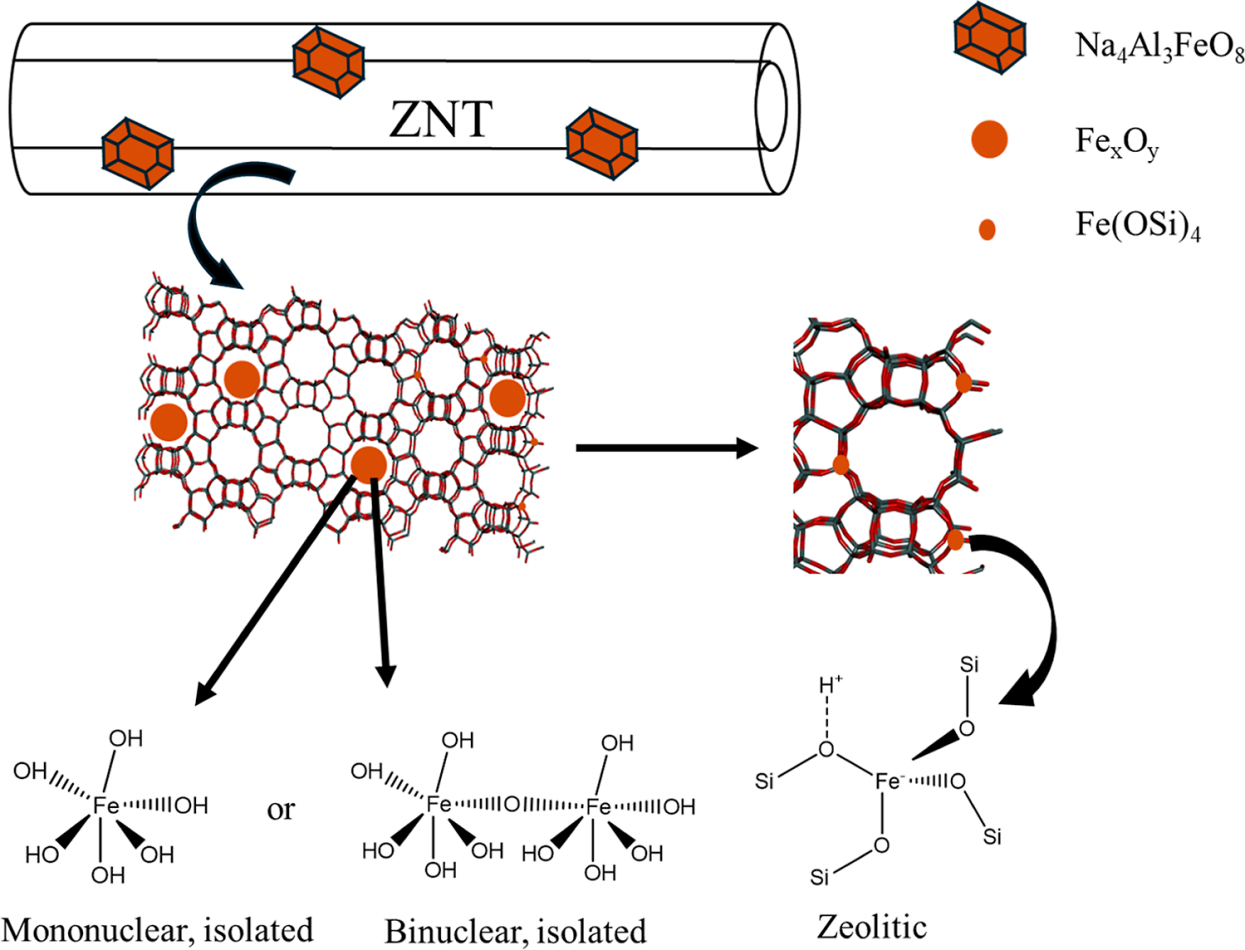
Cartoon of Proposed Fe Species Observed in TBA–Al/Fe–ZNT-*x*

TBA–Al/Fe–ZNT samples were characterized using DRUV–vis spectroscopy, shown in Figure 3B. For most Fe-zeolites, nonframework Fe is typically present as charge balancing, cationic Fe, pore occluded isolated oxide species (mononuclear or binuclear), or bulk oxides located on the exterior surface of the zeolite.^29^ For the nanotubes, the exterior surface could be either inside the tube’s mesopore or the exterior surface of the tube. The following bands are observed in TBA–Al/Fe–ZNT-1 and -2 : 210, 265, 320, and 390 nm, with bands increasing in intensity with increasing Fe loading. TBA–Al/Fe–ZNT-4 showed three bands located at 210, 265, and 320 nm, and TBA–Al/Fe-ZNT-8 showed two bands located at 210 and 265 nm. The bands located at 210 and 265 nm (present in all TBA–Al/Fe–ZNT samples) are assigned to isolated Fe species,^34,35^ which could consist of zeolitic Fe, or cationic Fe ([Fe(OH)_2_]^+^ or [Fe(OH)]^2+^) balancing framework Al. It is possible that the two bands located at 210 and 265 nm are both associated with zeolitic Fe, as the molecular orbital scheme predicts two charge transfer transitions for the same isolated Fe^3+^.^36,37^ The band located at 320 nm is still considered isolated Fe, which could consist of confined extra-framework Fe-oxide species, denoted as Fe_*x*_O_*y*_.^34^ These isolated confined species are typically pore occluded, in the 12-membered ring (12-MR) of BEA* or the 10-MR of MFI, and can consist of either mononuclear or binuclear species.^34,35,38,39^ At low Fe loadings (TBA-Al/Fe-ZNT-8), only two bands are observed, suggesting that only zeolitic and cationic Fe are present. When the Fe loading is increased to Al/Fe = 4, a small shoulder, assigned to Fe_*x*_O_*y*_ clusters, appears. With further increase in Fe loading (Al/Fe = 2–1) an additional band, located at 390 nm, is observed and is likely a non-zeolitic Fe-oxide species. This band correlates well to the appearance of the Bragg peak at ∼20.1° 2θ in Figure 3A, which was indexed as Na_4_Al_3_FeO_8_ [0 1 1] and [1 0 1]. A simplified cartoon of the proposed Fe species is shown in Scheme 1.

The ^29^Si MAS NMR spectra for TBA-Al-ZNT, TBA-Al/Fe-ZNT-*x*, and TBA-Al/B-ZNT-*x* are mostly similar, with the largest peak corresponding to the Q^4^ species Si-(OSi)_4_ and a smaller peak representing the Q^3^ species, which are either Si-(OSi)_3_(OAl) or Si-(OSi)_3_(OH). Framework Si-(OSi)_3_(OFe) or Si-(OSi)_3_(OB) will also show peaks in the Q^3^ region like Si-(OSi)_3_(OAl), where the more electronegative Fe^3+^ or B^3+^ would shift the Q^3^ peak downfield. The upfield shift was observed in the Fe and B samples (Table S3), suggestive of isomorphous substitution; however, the convolution of Si-(OSi)_3_(OH) in the Q^3^ region and error associated with peak deconvolution make this assignment tentative. The deconvoluted spectra and calculated Si/(Al + T) ratios are shown in Figure S11. Due to the large number of surface Si–OH groups associated with the single unit-cell thick ZNT wall, the calculated Si/(Al + T) ratios are likely underestimated. ICP-OES was performed on the TBA derived ZNT samples, and these data are shown in Table 3 (Table S4 for mass composition).

**Table 3:** ICP-OES Results for TBA–Al–ZNT, TBA–Al/Fe–ZNT-*x*, and TBA–Al/B–ZNT-*x*

sample	Si/Al	Si:(Al + T)	Si/T	Al/T	Na/Al
TBA-Al-ZNT	11.4				0.01
TBA-Al/Fe-ZNT-1	15.5	9.8	27	1.7	
TBA-Al/Fe-ZNT-2	14.0	10.4	40	2.9	
TBA-Al/Fe-ZNT-4	13.2	11.4	82	6.2	
TBA-Al/Fe-ZNT-8	10.2	9.00	78	7.65	
TBA-Al/B-ZNT-1	11.6	11.0	205^a^	17.6	
TBA-Al/B-ZNT-2	11.6	11.0	206^a^	17.6	
TBA-Al/B-ZNT-4	14.1	13.2	205^a^	14.5	
TBA-Al/B-ZNT-8	10.7	10.2	225^a^	21.1	

a Assumes B is incorporated at the detection limit of the ICP-OES analysis.

**Figure 4:**
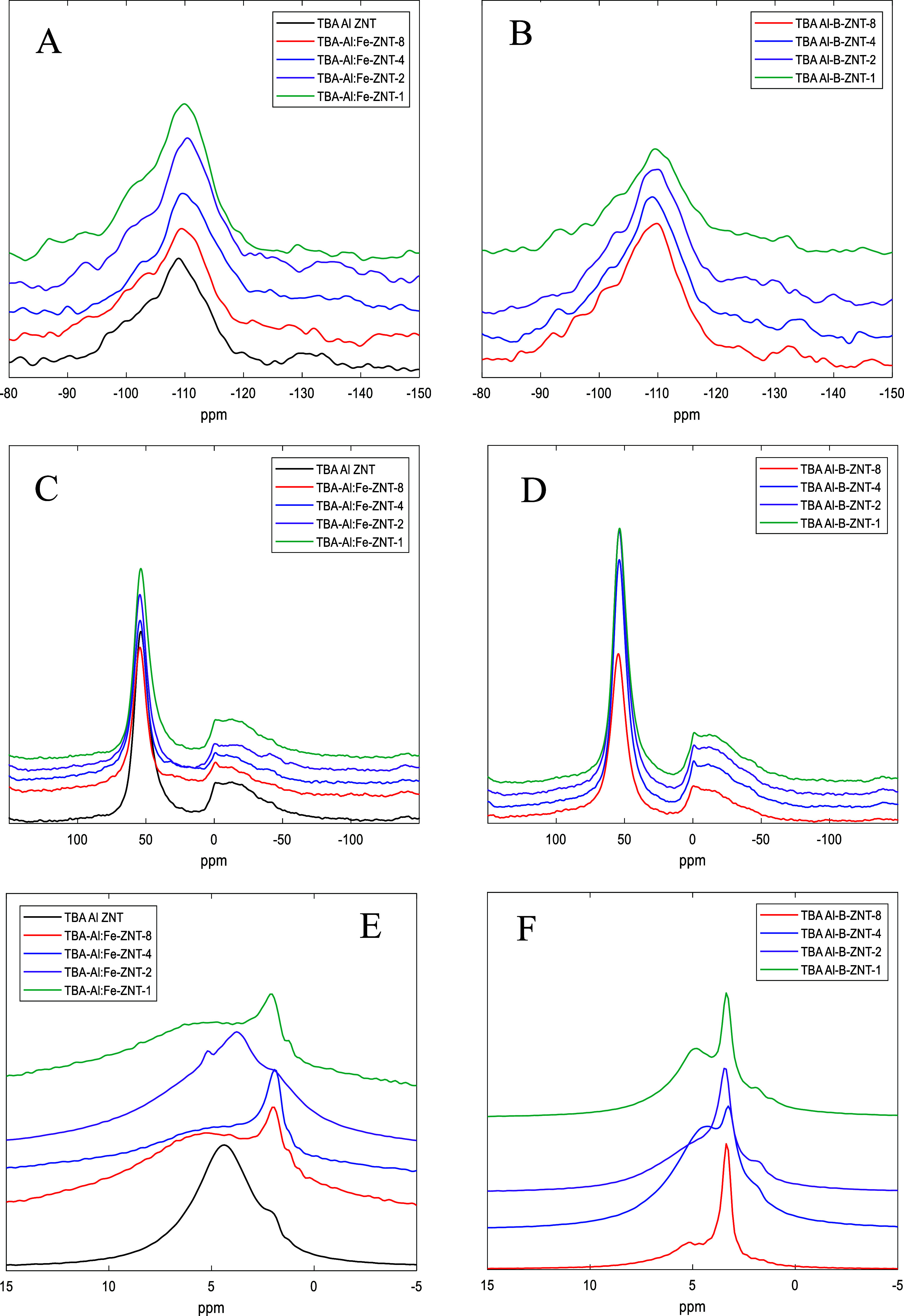
^29^Si MAS NMR spectra of: (A) TBA Al/Fe–ZNT-*x*, (B) TBA Al/B-ZNT-*x*; ^27^Al MAS NMR spectra of: (C) TBA Al/Fe–ZNT-*x*, (D) TBA Al/B-ZNT-*x*; ^1^H MAS NMR spectra of: (E) TBA Al/Fe–ZNT-*x*, (F) TBA Al/B–ZNT-*x*.

^27^Al MAS NMR (Figure 4C,D) shows peaks in two distinct regions. The peak located at ∼55 ppm is assigned to tetrahedral, zeolitic Al and the peak located at ∼0 ppm is assigned to octahedral, extra framework Al (EFAl). Previous work showed no such EFAl peak in NaH-ZNT,^15^ meaning that the EFAl could be arising from the difference in Na^+^-content between NaH-ZNT and TBA-Al-ZNT, where the more H^+^-rich zeolite is more prone to framework-Al removal. Another possibility is the potential difference in Al distribution associated with the organic/inorganic cation ratio in the gel, where the TBA synthesis could be giving more Al–Al pairs,^22−25^ which could be prone to framework removal (dealumination) upon calcination, as the local Al-content is higher than single site Al. Similar results have been observed for conventional MEL elsewhere.^25^ To test this hypothesis, two ion exchanged samples were prepared. TBA-SDA-ZNT was ion exchanged with Na^+^ and Na–SDA–ZNT was ion exchanged with NH_4_^+^. After calcination, octahedral Al was observed in both samples (Figure S12E,F) suggesting that the EFAl is produced both in the pure protonic ZNT, with hypothesized single Al sites (NaH–ZNT–NH_4_^+^-ex) and in the mixed Na/H form of ZNT with hypothesized paired Al sites (TBA–Al–ZNT–Na^+^-ex).

^1^H MAS NMR spectra (Figure 4E,F) of the ZNTs (under ambient conditions) show the main three signals. The furthest downfield signal (5.3–4.4 ppm) is assigned to H-bonded bridged hydroxyls (SiOHAl), where the proton is H-bonding with either adsorbed H_2_O or framework oxygen.^32,40,41^ BAS that H-bond with framework oxygen is suggestive of sitting in a 5-MR or 6-MR secondary building unit.^40,42^ TBA–Al/Fe–ZNT-2 shows a peak at 5.2 ppm and a broad peak at 3.7 ppm. The broadness of the peak at 3.7 ppm is suggestive of H-bonded BAS, described above, whereas the peak at 5.2 ppm is assigned to H-bonded SiOH.^41^ The next peak, present in all samples between 2.1 and 1.9 ppm is assigned to external SiOH.^32,40−42^

^11^B MAS NMR was conducted on the TBA–Al/B–ZNT-*x* samples to assess the state of B in the zeolite. Even with rotational echo, which is typical for B zeolites to amplify the inherently weak signal, B was not detected by NMR, suggesting that a large fraction of B remains in the mother liquor after crystallization. Therefore, B^3+^ in the gel appears to primarily modulate the proper solubility conditions to crystallize ZNT, while remaining in the liquid phase after crystallization. Contrary to Fe^3+^, B^3+^ is moderately soluble in a base, which may explain why more B remains in the liquid phase after crystallization. The Na^+^ in the TBA gels is sufficient to balance the charge of BO_2_^–^ (Na_2_O/B_2_O_3_ = 2.5) meaning that B likely remains dissolved as NaBO_2_. The solids yield [mass ZNT/mass SiO_2_ + T_2_O_3_ (gel)] of TBA-Al ZNT versus TBA-Al/B-ZNT-1 is 30 and 22% respectively, further suggesting that B remains in the liquid phase after crystallization. These results show that nanotubes will crystallize when the following gel composition parameter is satisfied: 1 < Al/*T* < ∞ when Si/(Al + T) = 30. For example, a ZNT sample was obtained using a ternary T atom system where Al/Fe/B (i.e., Al/(Fe + B) was 2:1:1 and Si/(Al + Fe + B) = 30 (Figure S13).

### Catalytic Testing Via Tandem CO_2_ to Methanol to Aromatics Reaction

**Figure 5:**
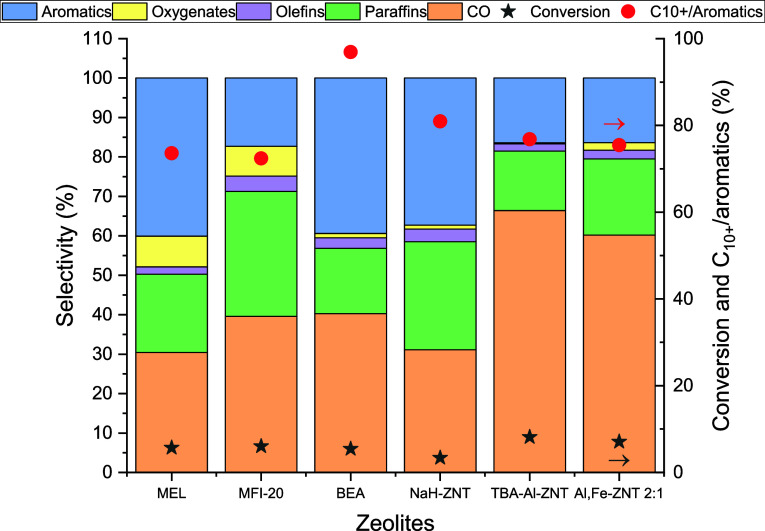
CO_2_ conversion and product selectivity using various zeolite catalysts where ZnZrO_*x*_/zeolite = 1:2 and Zn/Zr = 6. *P* = 600 psig, temperature = 320 °C, WHSV = 7200 mL g_cat_^–1^ h^–1^.

Zeolites (e.g., H-ZSM-5) have been studied in tandem with ZnO–ZrO_2_ for conversion of CO_2_ to aromatics, where CO_2_ is first converted to methanol (MeOH) over the ZnO–ZrO_2_ catalyst, while MeOH is converted to aromatic hydrocarbons over the zeolite phase. This tandem reaction is chosen to provide evidence of zeolite Brønsted acidity in a gas-phase, high-temperature reaction, since the reaction is currently routinely run in our laboratory. Nezam et al. observed that the transport length between the zeolite and metal-oxide phase is critical to improving CO_2_ to aromatics yield, which was controlled by testing different particle sizes of H-ZSM-5.^17,18,43^ In this regard, we hypothesize that the 1D structure of ZNTs will facilitate efficient transfer of methanol from the metal oxide phase to the zeolite phase and that the thin zeolite domains and large mesopores in the ZNTs should lead to production of larger than average aromatics. The catalytic activity of the NaH–ZNT, TBA–Al–ZNT, and TBA–Al/Fe–ZNT-*x* samples (all mixed with ZnO–ZrO_2_) was tested for tandem CO_2_ hydrogenation/methanol conversion and compared to various common zeolite acid catalysts. CO_2_ conversions and product selectivities for the different zeolites tested are shown in Figure 5.

As compared to MFI of similar Si/Al, the selectivity to aromatics is higher when ZNTs were used. This may be due to fewer transport barriers between the BAS of zeolite and the methanol producing sites of the metal oxide when using ZNTs compared to bulk 3D MFI. Many of these aromatics are C_10+_, which can be ascribed to the large pore domains akin to the BEA* phase comprising the outer nanotube wall. Conventional BEA* shows almost all C_10+_, which is likely due to the product selectivity associated with the large 12-ring pores, where larger aromatic products are permitted to diffuse into the 3D zeolite phase. In contrast, these heavily alkylated aromatics are generally too large to desorb from the medium 10-ring pore opening of H-ZSM-5 and thus are not produced to the same degree over medium pore zeolite catalysts. Like BEA*/ZnO–ZrO_2_, NaH–ZNT/ZnO–ZrO_2_ also shows a very low oxygenate selectivity. Larger pore size and lower diffusion restrictions likely allow for further methylation of hydrocarbon products, allowing for efficient conversion of oxygenates (e.g., methanol) to hydrocarbons.^44^ The higher efficiency of the transport of the oxygenates over ZNT is also supported by the observation that NaH–ZNT/ZnO–ZrO_2_ shows lower oxygenate selectivity as compared to 2D-MEL/ZnO–ZrO_2_, implying that the transport of the oxygenates, especially methanol and DME, is slower than in the case of ZNT.

TBA–Al–ZNT and TBA–Al/Fe–ZNT-*x* also show conversion to aromatics; however, there is a significant increase in the CO selectivity, which may be ascribed to the different oxides present in these catalysts (EFAl and Fe-oxides). CO is a common side product in the CO_2_ to aromatics reaction and is generated via the reverse water–gas shift reaction of CO_2_ and H_2_. In TBA–Al–ZNT, higher CO selectivity is observed compared to NaH ZNT. In a previous study, Shah et al. observed that isomorphous substitution of Al with Fe in the framework improved aromatics selectivity in 3D ZSM-5.^45^ Here, with TBA–Al/Fe–ZNT-*x*, extra framework Fe species (bulk oxides or isolated oxides) may contribute to an increase in CO selectivity, as Fe-oxides are known to be active toward the RWGS reaction.^46^ However, a slight decrease in CO selectivity was observed with a slight increase in the paraffin selectivity as well as oxygenates, with an increase in Fe content. This potentially could be attributed to the Fischer–Tropsch conversion of produced CO to hydrocarbons^47^ as well as conversion of CO_2_ to oxygenates^48^ over Fe-oxide phases. Furthermore, because the onset of deactivation of ZSM-5 was shown to take 21 d under these conditions,^45^ deactivation of ZNT in the 12 h runs used here is unlikely. These results suggest that the new ZNTs synthesized here, like the original ZNTs published previously,^15,49^ have conventional zeolitic Brønsted acidity and offer products distributions that can be rationalized based on the zeolite pore structure.

## Conclusions

The scope of parameters for single walled zeolite nanotube synthesis was explored, and a new TBAOH mediated synthesis route to ZNT was developed. ZNTs were obtained across a range of gel compositions where Na^+^/TBA^+^ ranged from 0.4 to 0.6, Al/T from 1 to 8 (T = Fe^3+^ or B^3+^), and Si/(Al + T) was held constant at 30, resulting in zeolite phases ranging from 11 to 16 in SAR. Mesoscale differences in nanotube arrangement depended on the Na^+^-content of the gel, consistent with previous work on MFI nanosheets. It is hypothesized that the Na^+^-content may also change the zeolite’s Al-distribution; however, more work is needed to rigorously test this hypothesis. Elemental analysis provided evidence of Fe incorporation into the zeolite. In contrast, B was not significantly incorporated, being undetected by both ICP-OES and ^11^B MAS NMR. The incorporated Fe^3+^ species were characterized with DRUV–vis spectroscopy and varied with the Fe-content in the gel. When probed with the CO_2_ to aromatics reaction, the ZNTs showed comparable aromatics selectivities to HMEL and HBEA* with similar SARs. TBA-derived nanotubes (with and without Fe) showed higher CO selectivities, which is ascribed to the EFAl and extra-framework Fe-oxides present, promoting the reverse water gas shift reaction in parallel with methanol formation.

## Supporting Information Available

Table of the sample nomenclature used throughout this work, additional characterization on ZNT samples includes N_2_ isotherms and TEM images of solvent exposed, degraded ZNTs, in situ XRD patterns (up to 800 °C), the N_2_ physisorption isotherms after the in situ XRD experiment are shown, characterization of uncalcined Na-SDA-ZNT and extracted Na–SDA–ZNT (treated with methanol/formic acid) is reported, next is the XRD and N_2_ physisorption characterization on the mixed Na/TBA ZNT series, followed by the XRD patterns of TBA–Al/Fe–ZNT-1 showing densification to MEL with time, the NH_3_ TPD curves and summarized data are shown for all ZNT samples, characterization, showing the effect of pelletization as well as the deconvoluted ^29^Si MAS NMR spectra are shown, lastly is the raw ICP-OES data, followed by characterization of the ion exchanged ZNT samples and the characterization on TBA-Al/Fe/B-ZNT-2:1:1 (PDF)
