# Supplementary material for: Synthesis Route to Single-Walled Zeolite Nanotubes Enabled by Tetrabutylammonium Hydroxide
Source: ACS Mater Au. 2024 Jul 11;4(5):523–36. doi: 10.1021/acsmaterialsau.4c00030 (PMC11393933; doi:10.1021/acsmaterialsau.4c00030)
Supplement: Supplementary file 1 — mg4c00030_si_001.pdf [file mg4c00030_si_001.pdf]

Supporting Information for:

A New Synthesis Route to Single-Walled Zeolite Nanotubes Enabled by TBAOH

Anthony Vallace, Dhrumil R. Shah, Enerelt Burentugs, Atticus J. Tucker, Ashley E. Cavanagh, Christopher W. Jones*

School of Chemical & Biomolecular Engineering, Georgia Institute of Technology, 311 Ferst Drive, Atlanta, Georgia 30332, United States

Email: cjones@chbe.gatech.edu

Table S1. Zeolite nanotube sample nomenclature

Sample name	Description
Na-SDA-ZNT	NaOH route, uncalcined ZNT
TBA-SDA-ZNT	TBA route, uncalcined ZNT
NaH-ZNT	NaOH route, calcined ZNT
NaH-ZNT-NH_4^+-ex	Na-SDA-ZNT→ 3X NH_4^+ exchanged, calcined after exchange
TBA-Al-ZNT-Na^+-ex	TBA -SDA-ZNT→ 3X Na^+ exchanged, calcined after exchange
Na:TBA-Al-ZNT-*x*	Mixed NaOH/TBAOH gels where *x* = molar ratio of Na:TBA
TBA-Al-ZNT	TBAOH route, calcined ZNT, Al is only trivalent T-atom
TBA-Al:Fe-ZNT-*x*	TBAOH route, calcined ZNT, *x* = molar ratio of Al to Fe in gel
TBA-Al:B-ZNT-*x*	TBAOH route, calcined ZNT, *x* = molar ratio of Al to B in gel

Table S2. Textural properties of ZNT samples

Sample	BET Surface Area (m^2/g)	Total pore volume (cc/g)	Mesopore width (nm)
NaHZNT	1160	1.76	2.4
TBA-Al-ZNT	1250	1.37	2.3
Na:TBA-Al-ZNT-0.09	715	0.90	2.1
Na:TBA-Al-ZNT-0.15	1210	1.35	2.1
Na:TBA-Al-ZNT-0.31	995	1.21	2.2
Na:TBA-Al-ZNT-0.58	1265	1.36	2.6
Na:TBA-Al-ZNT-1.7	1030	1.28	2.2
Na:TBA-Al-ZNT-2.2	1130	1.41	2.7
Na:TBA-Al-ZNT-5.2	1290	1.59	2.7
TBA Al:Fe ZNT-1	1160	1.31	2.6
TBA Al:Fe ZNT-2	1130	1.40	2.2
TBA Al:Fe ZNT-4	1160	1.36	2.1
TBA Al:Fe ZNT-8	1300	1.29	2.1
TBA Al:B ZNT-1	1350	1.31	2.1
TBA Al:B ZNT-2	1080	1.26	2.5
TBA Al:B ZNT-4	1155	1.23	2.5
TBA Al:B ZNT-8	1065	1.26	2.1

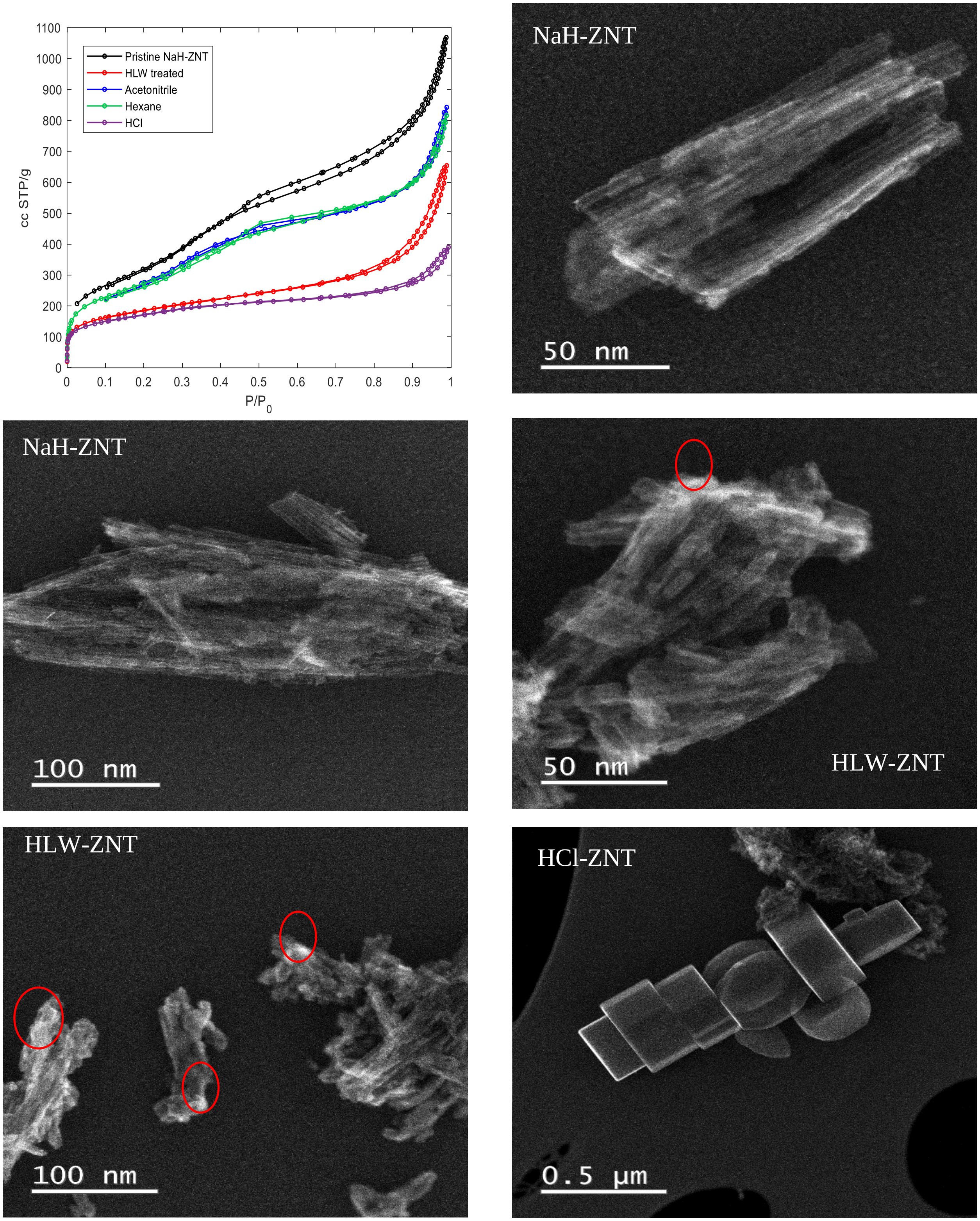

Figure S1. N_2 physisorption isotherms of HLW-ZNT, acetonitrile-ZNT, hexane-ZNT. TEM images of pristine Na-H-ZNT, HLW-ZNT, and HCl-ZNT. (red circles denote debris)

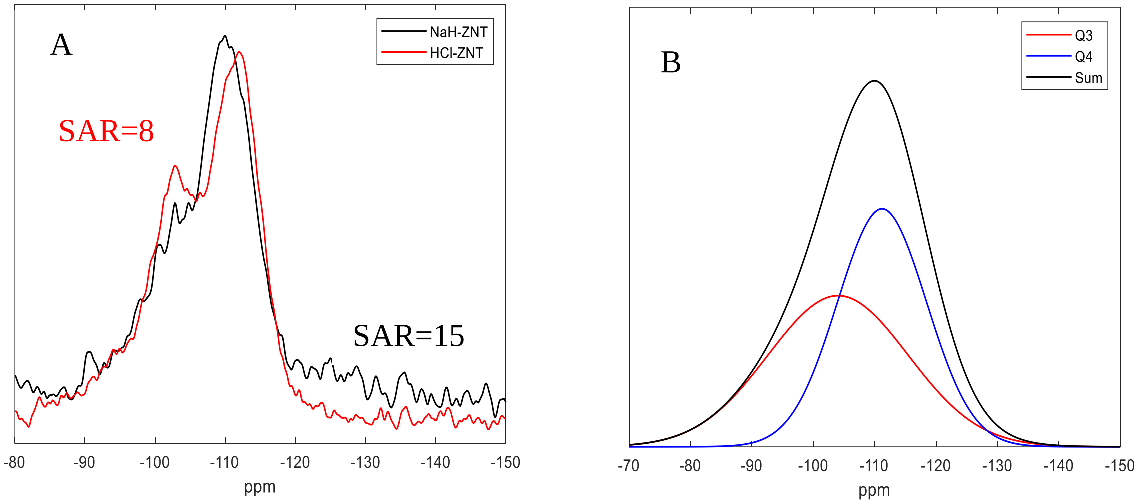

Figure S2: A) ^{29}Si MAS NMR spectra of NaH-ZNT (black) and HCl-ZNT (red) and B) deconvolution of HCl-ZNT.

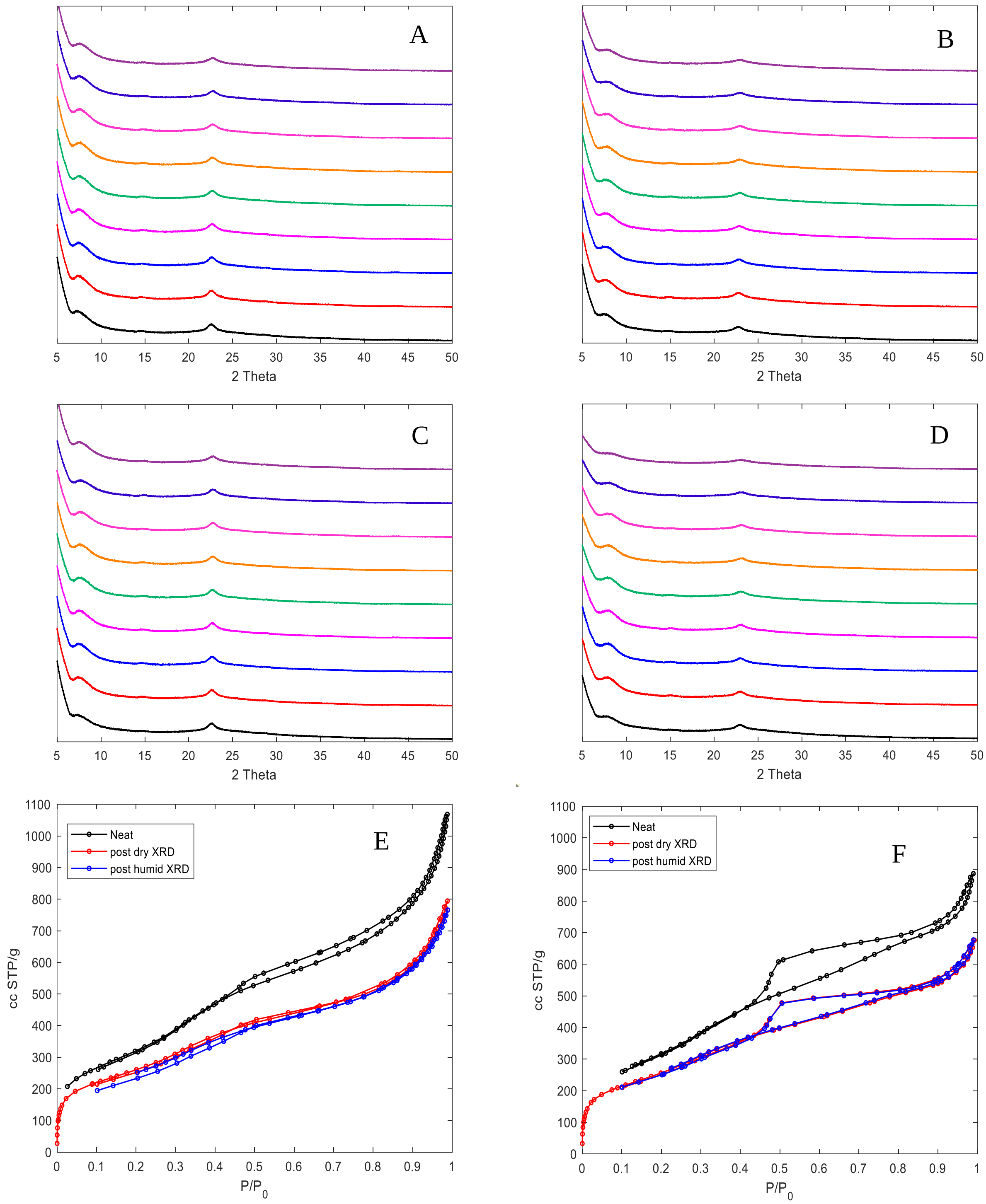

Figure S3. In situ XRD patterns of NaH-ZNT under A) dry N_2 B) humid N_2. TBA-Al-ZNT under C) dry N_2 and D) humid N_2 (From bottom to top: 25 °C, 150 °C, 200 °C, 300 °C, 400 °C, 500 °C, 600 °C, 700 °C, 800 °C. Dew point = 15 °C for humid scans). N_2 physisorption isotherms of NaH-ZNT (E) and TBA-Al-ZNT (F) before and after in situ XRD experiments (red: dry N_2 used, blue: humid N_2 used).

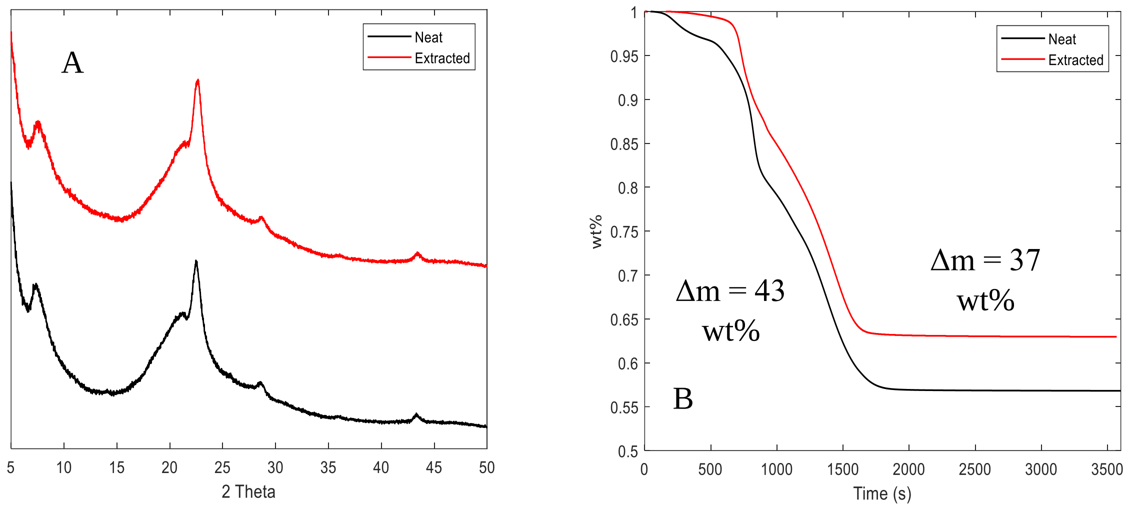

Figure S4. A) XRD patterns and B) combustion TGA of Na-SDA-ZNT before and after two extractions with methanol/formic acid

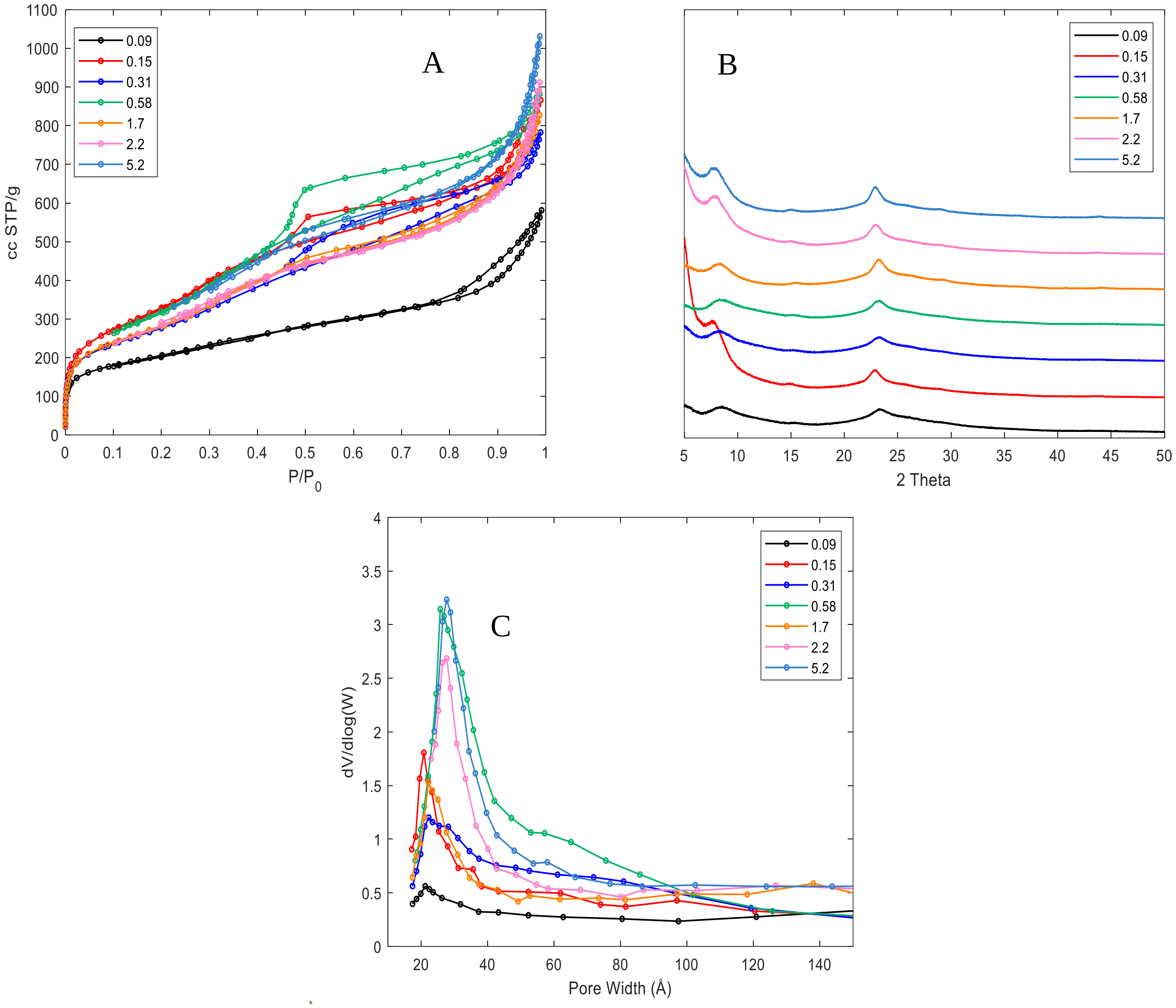

Figure S5. A) N_2 physisorption isotherms, B) XRD patterns of Na:TBA-Al-ZNT-*x* and CdV/dlog(W) pore size distribution. Refer to **Table 1** for [SDA+Na_2O+TBA_2O]:SiO_2 ratios.

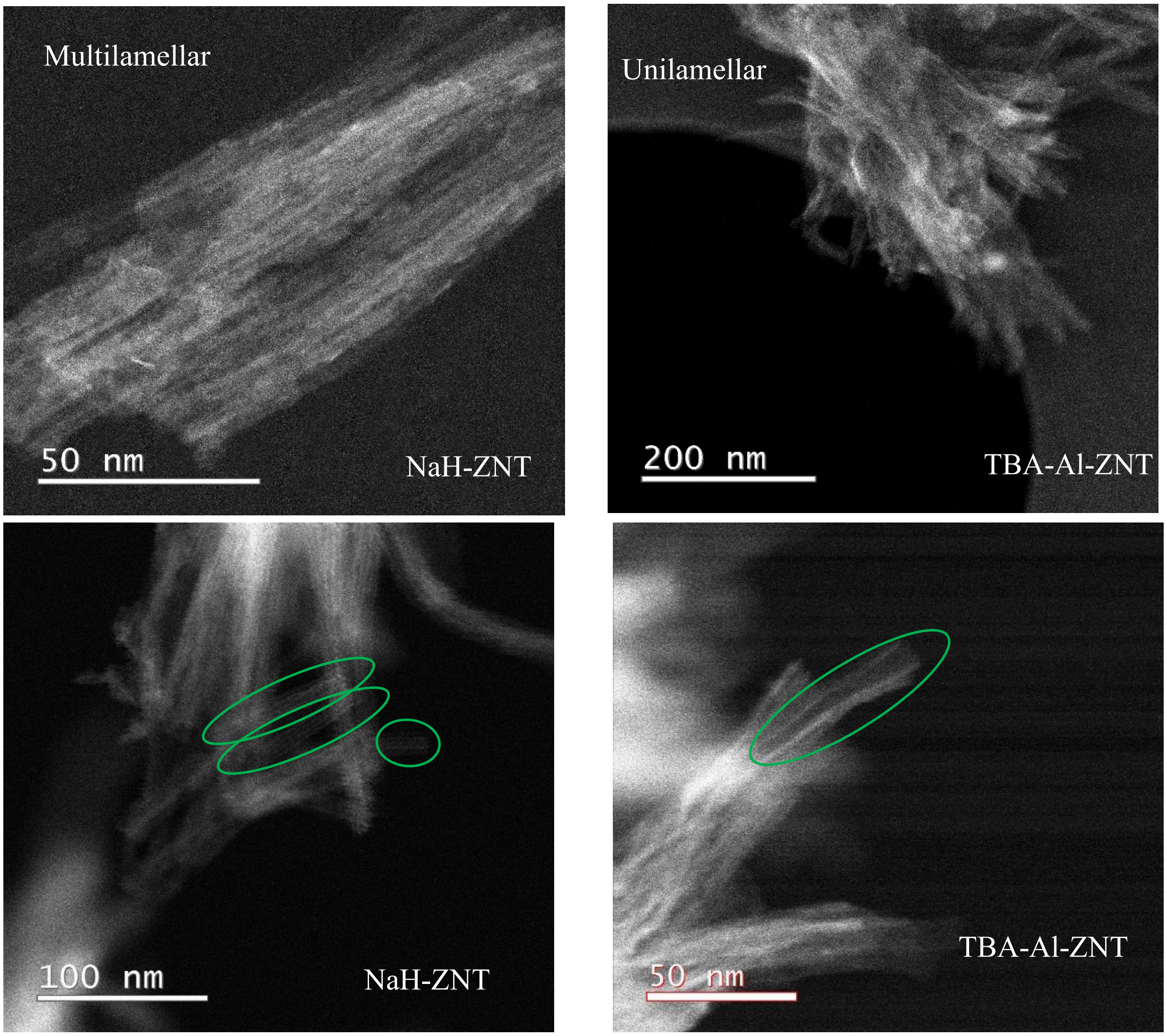

Figure S6. TEM images of NaH-ZNT and TBA-Al-ZNT. Top two images showing unilamellar versus multilamellar orientation, bottom two images show individual nanotubes (green markings showing nanotube structure).

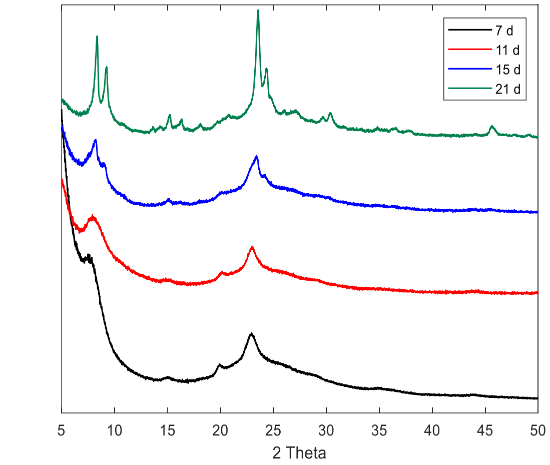

Figure S7. XRD patterns of TBA-Al:Fe-ZNT-1 synthesized at different crystallization times

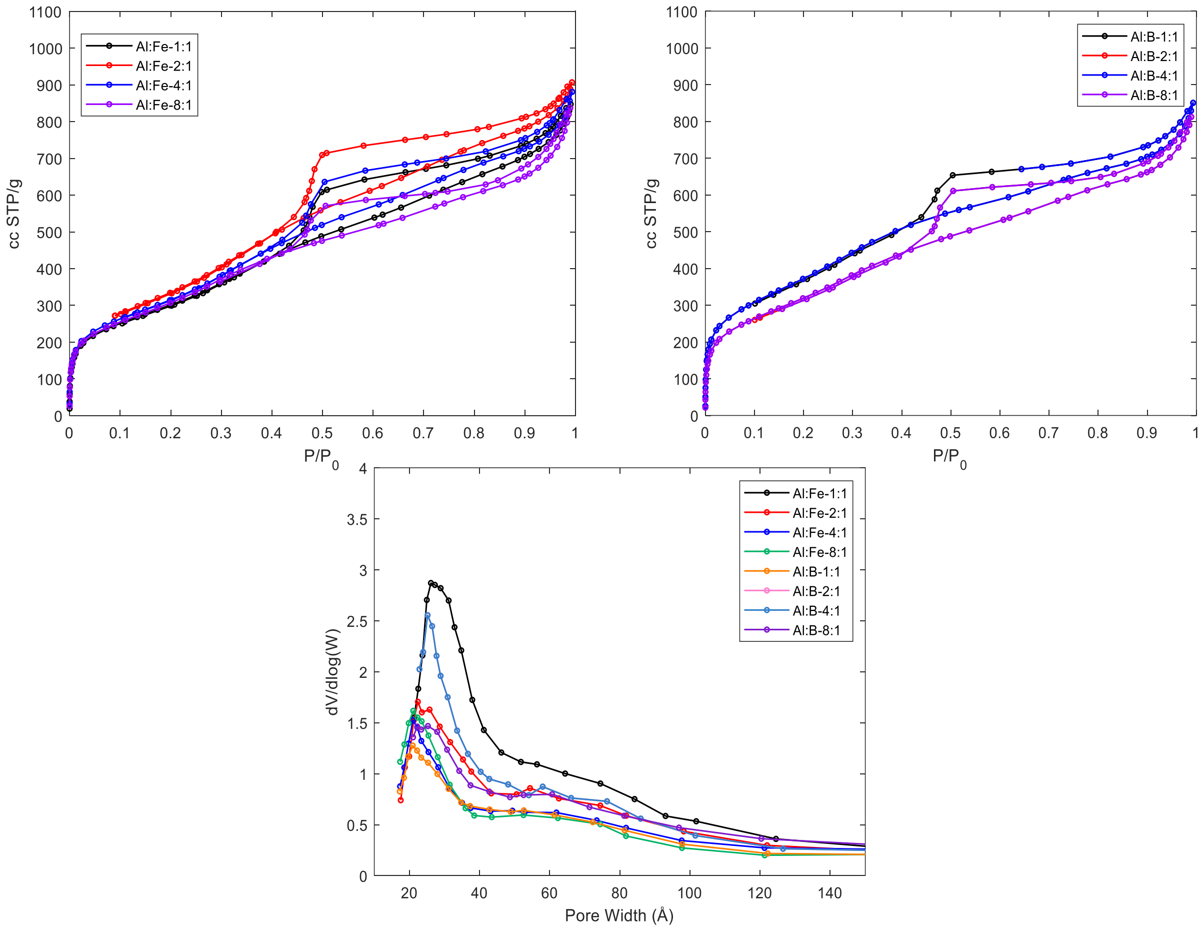

Figure S8. N_2 physisorption isotherms and dV/dlog(W) pore size distribution of TBA-Al:Fe-ZNT1-8 and TBA-Al:B-ZNT1-8

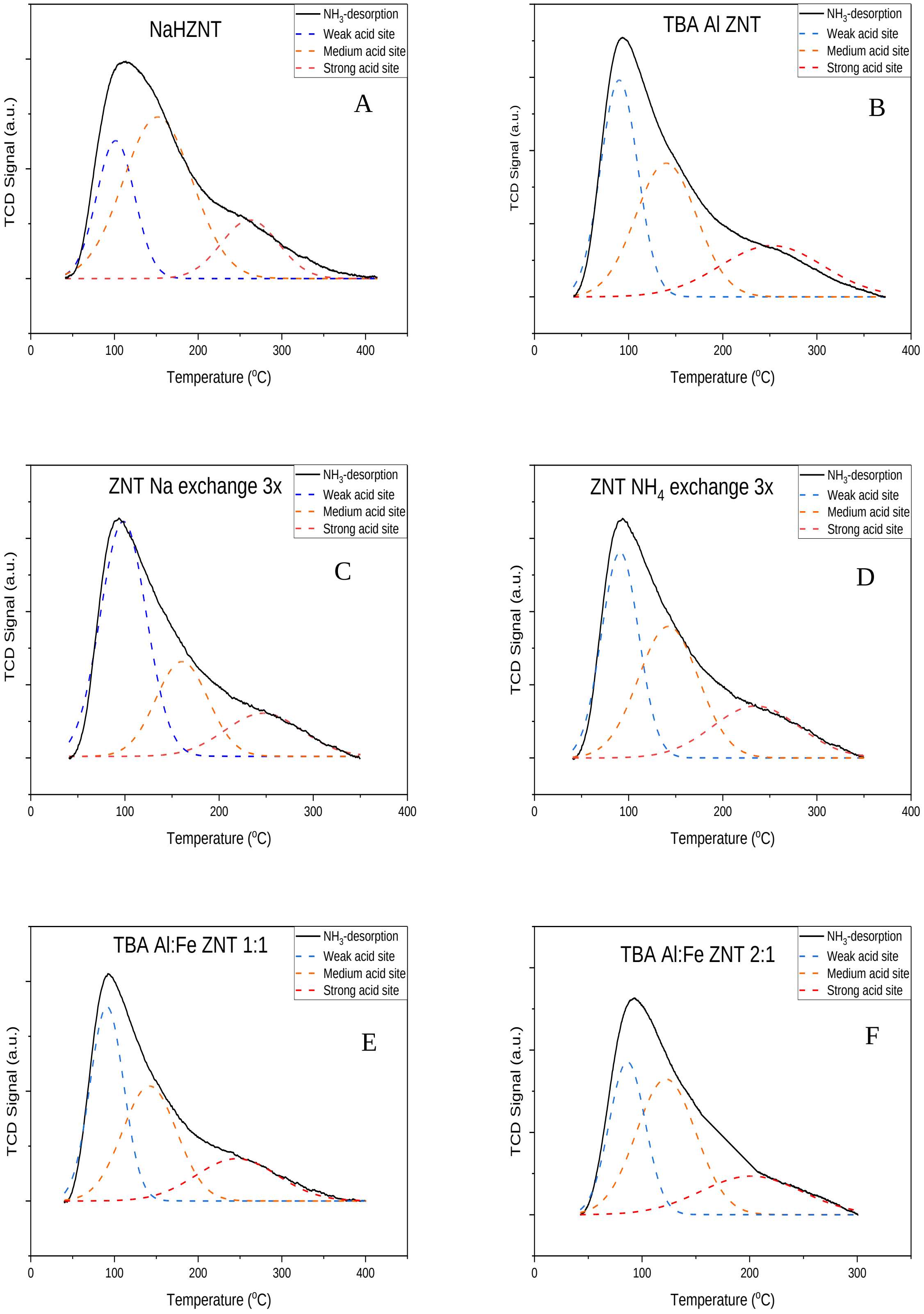
NaHZNT
A
TBA Al ZNT
B
ZNT Na exchange 3x
C
ZNT NH4 exchange 3x
D
TBA Al:Fe ZNT 1:1
E
TBA Al:Fe ZNT 2:1
F
NH3-desorption
Weak acid site
Medium acid site
Strong acid site
TCD Signal (a.u.)
Temperature (°C)
0
100
200
300
400

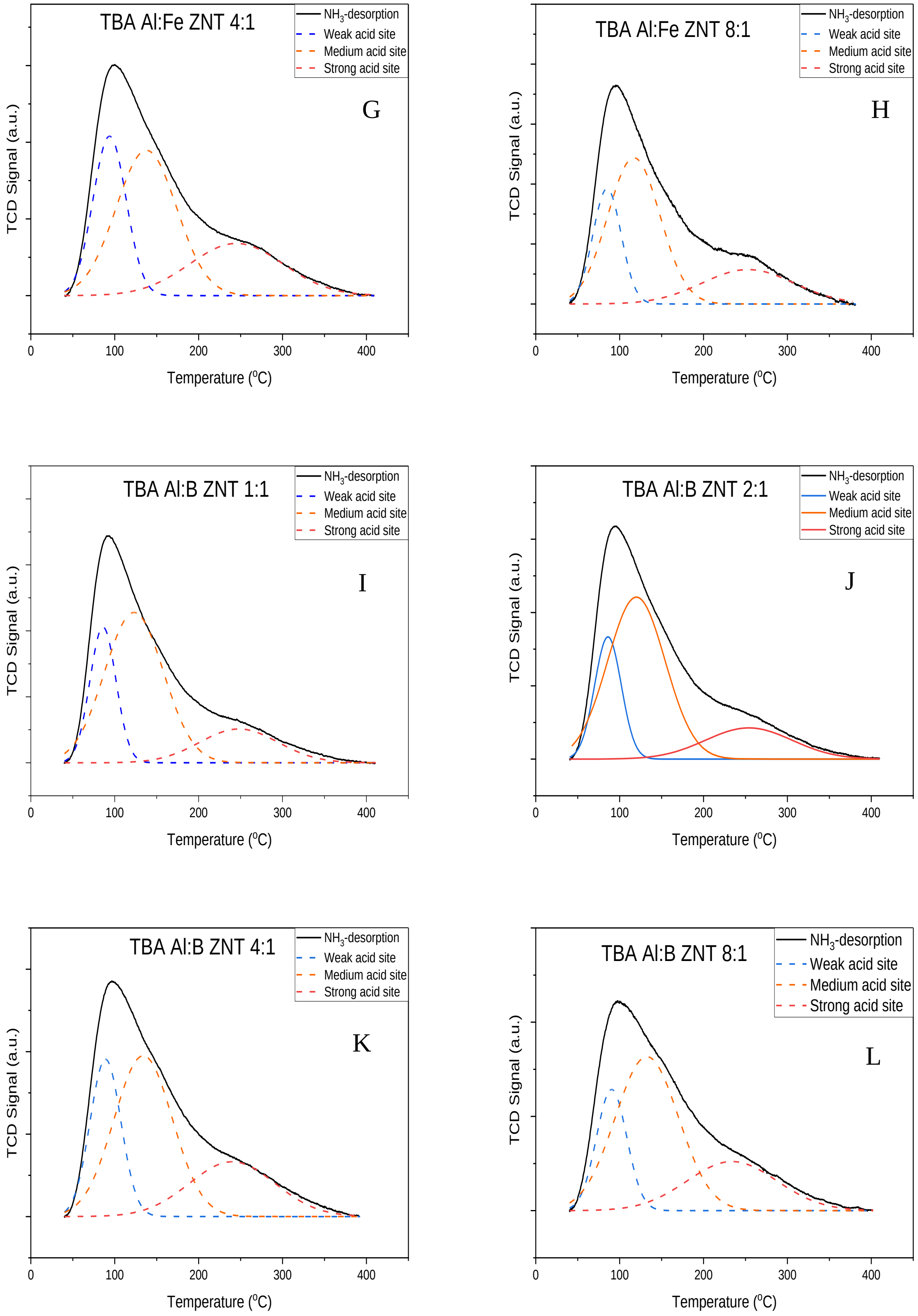
TBA Al:Fe ZNT 4:1
NH3-desorption
Weak acid site
Medium acid site
Strong acid site
G
TCD Signal (a.u.)
Temperature (°C)
TBA Al:Fe ZNT 8:1
NH3-desorption
Weak acid site
Medium acid site
Strong acid site
H
TCD Signal (a.u.)
Temperature (°C)
TBA Al:B ZNT 1:1
NH3-desorption
Weak acid site
Medium acid site
Strong acid site
I
TCD Signal (a.u.)
Temperature (°C)
TBA Al:B ZNT 2:1
NH3-desorption
Weak acid site
Medium acid site
Strong acid site
J
TCD Signal (a.u.)
Temperature (°C)
TBA Al:B ZNT 4:1
NH3-desorption
Weak acid site
Medium acid site
Strong acid site
K
TCD Signal (a.u.)
Temperature (°C)
TBA Al:B ZNT 8:1
NH3-desorption
Weak acid site
Medium acid site
Strong acid site
L
TCD Signal (a.u.)
Temperature (°C)
0
100
200
300
400

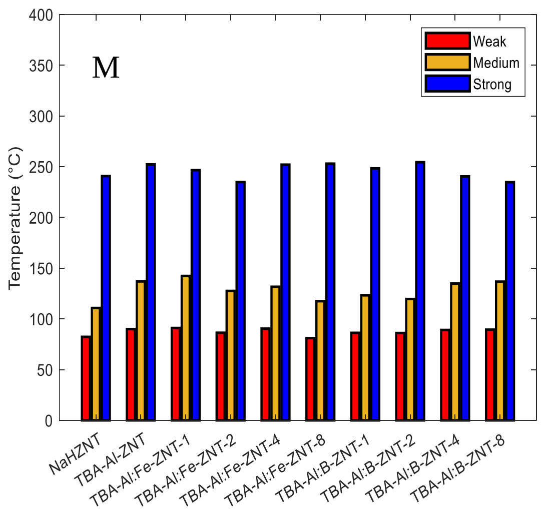

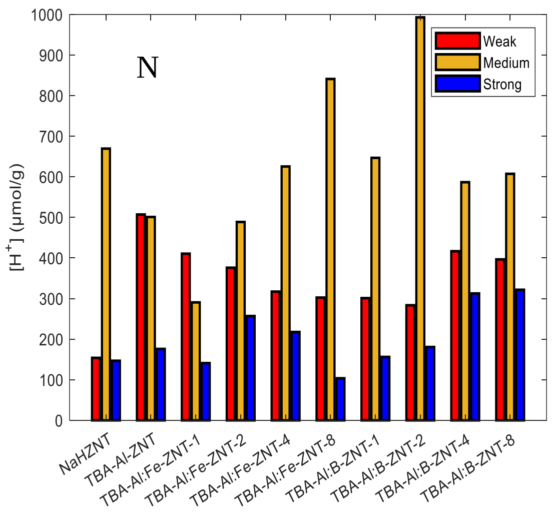

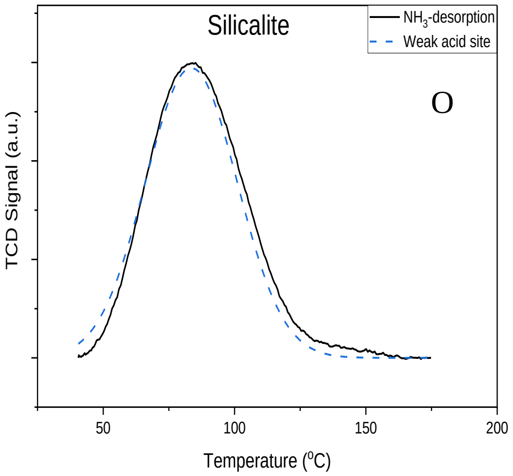

Figure S9. NH_3 TPD profiles of A) NaH-ZNT and B) TBA-Al-ZNT, C) Na and D) NH_4 exchanged Na-SDA-ZNT E-H) TBA-Al:Fe-ZNT-*x* and I-L) TBA-Al:B-ZNT-*x*; M) NH_3 desorption temperature and N) acid site concentrations of strong, medium , and weak acid sites; O) NH_3 TPD profile of silicalite (pure SiO_2 MFI)

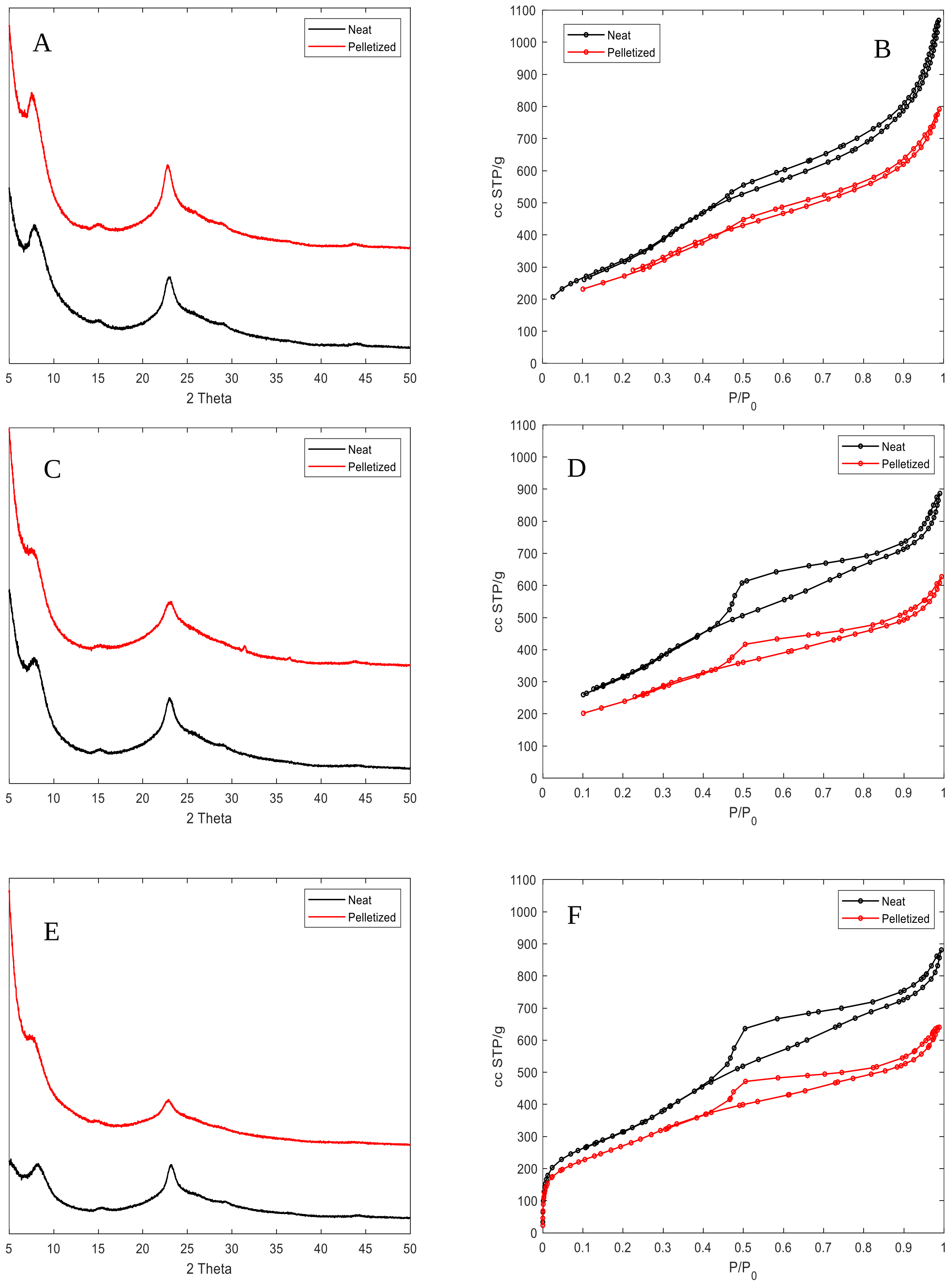

Figure S10. XRD patterns and N_2 physisorption isotherms of NaH-ZNT (A,B), TBA-Al-ZNT (C,D), and TBA-Al:Fe-ZNT-8 (E,F) before (black) and after (red) pelletization

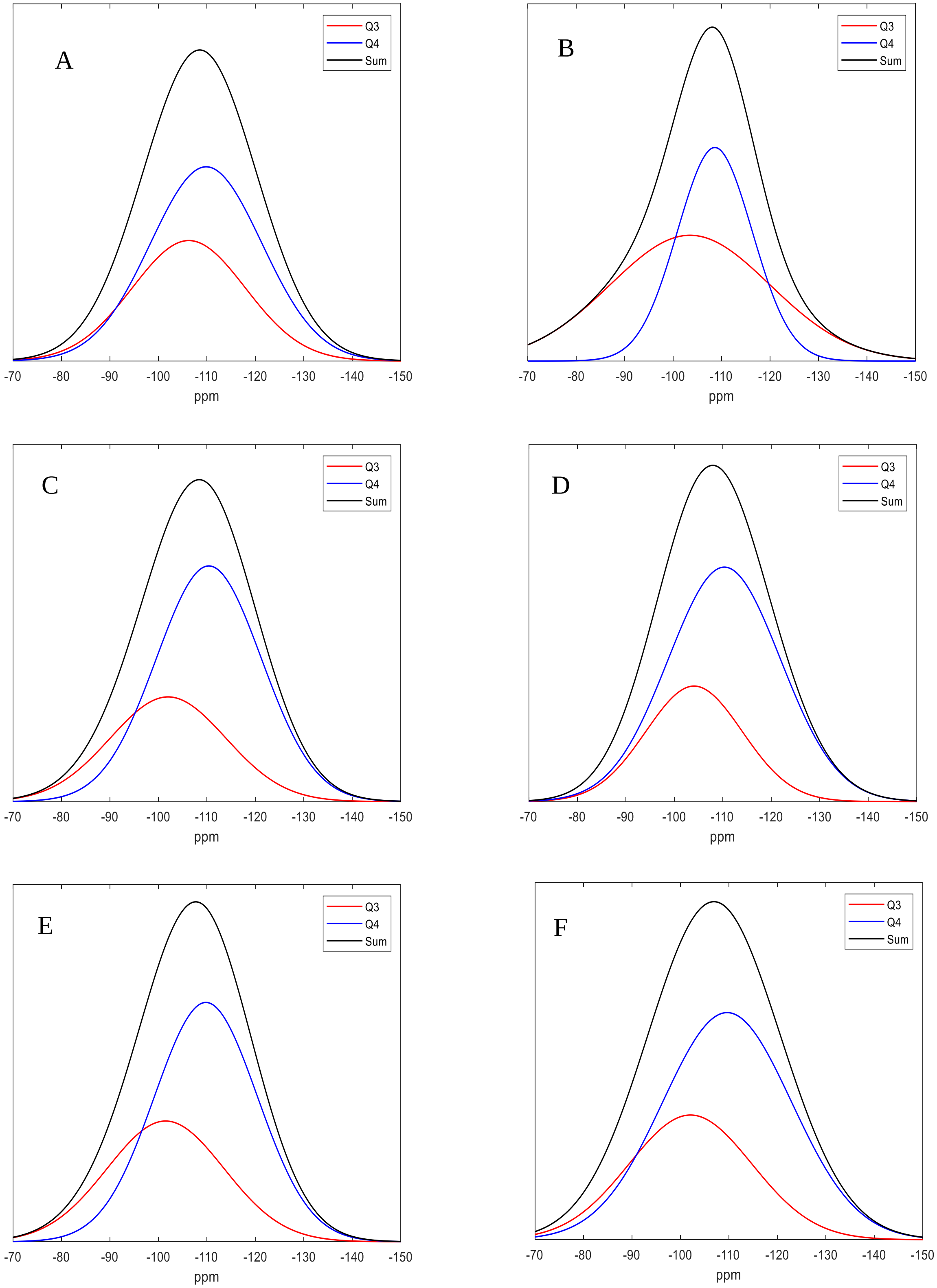
A
B
C
D
E
F
Q3
Q4
Sum
-70
-80
-90
-100
-110
-120
-130
-140
-150
ppm

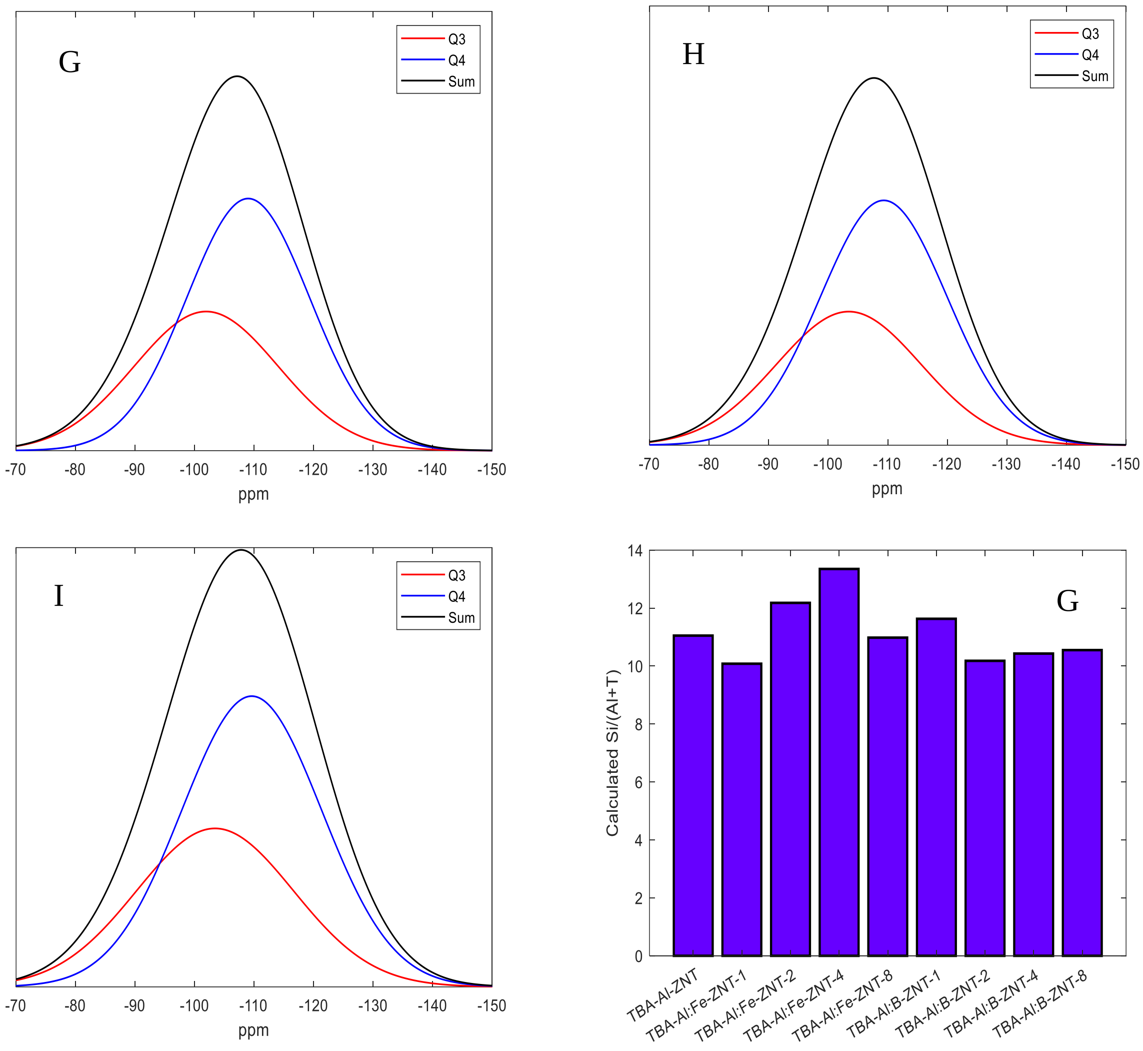

Figure S11. Deconvoluted ^{29}Si MAS NMR spectra of A)TBA-Al-ZNT; B-E) TBA-Al:Fe-ZNT-1, 2, 4, 8; F-I) TBA-Al:B-ZNT-1, 2, 4, 8; G) calculated atomic Si/Al of all TBA-ZNT samples from NMR results

Table S3. ^{29}Si MAS NMR Q4 and Q3 chemical shift values

Sample	Q^4 (ppm)	Q^3(ppm)
TBA-Al-ZNT	109.8	-106.2
TBA-Al:Fe-ZNT-1	-109.2	-104.8
TBA-Al:Fe-ZNT-2	-110.4	-102.0
TBA-Al:Fe-ZNT-4	-110.3	-104.1
TBA-Al:Fe-ZNT-8	-109.8	-102.7
TBA-Al:B-ZNT-1	-109.6	-102.7
TBA-Al:B-ZNT-2	-109.0	-101.9
TBA-Al:B-ZNT-4	-109.3	-103.4
TBA-Al:B-ZNT-8	-109.6	-103.4

Table S4. ICP-OES results for TBA derived ZNT samples

Sample	Si (wt%)	Al (wt%)	Na (wt%)	Fe (wt%)	B (wt%)	O (wt%)
TBA-Al-ZNT	40.5	3.41	0.028	-	-	56.1
TBA-Al:Fe-ZNT-1	41.1	2.54	-	3.04	-	53.3
TBA-Al:Fe-ZNT-2	38.3	2.62	-	1.90	-	57.2
TBA-Al:Fe-ZNT-4	39.0	2.82	-	0.944	-	57.2
TBA-Al:Fe-ZNT-8	37.2	3.50	-	0.950	-	58.4
TBA-Al:B-ZNT-1	39.8	3.29	-	-	0.075	46.8
TBA-Al:B-ZNT-2	41.1	3.38	-	-	0.077	55.4
TBA-Al:B-ZNT-4	40.4	2.75	-	-	0.076	56.8
TBA-Al:B-ZNT-8	41.5	3.73	-	-	0.071	54.7

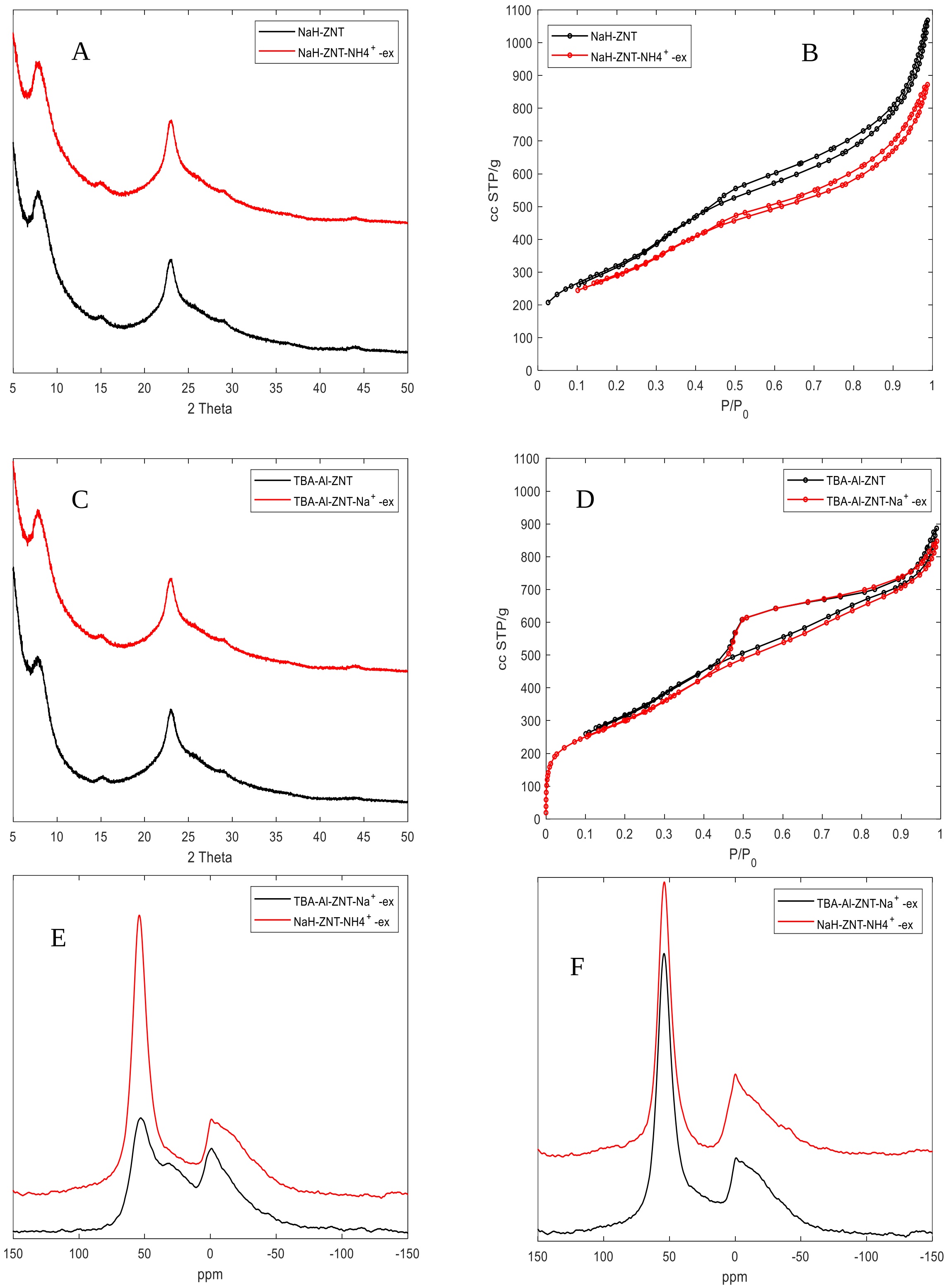

Figure S12. XRD patterns and N_2 physisorption isotherms of NaH-ZNT and NaH-ZNT-NH_4^+-ex (A, B) and TBA-Al-ZNT and TBA-Al-ZNT-Na^+-ex (C,D); ^{27}Al MAS NMR spectra of E) dried and F) hydrated NaH-ZNT-NH_4^+-ex and TBA-Al-ZNT-Na^+-ex

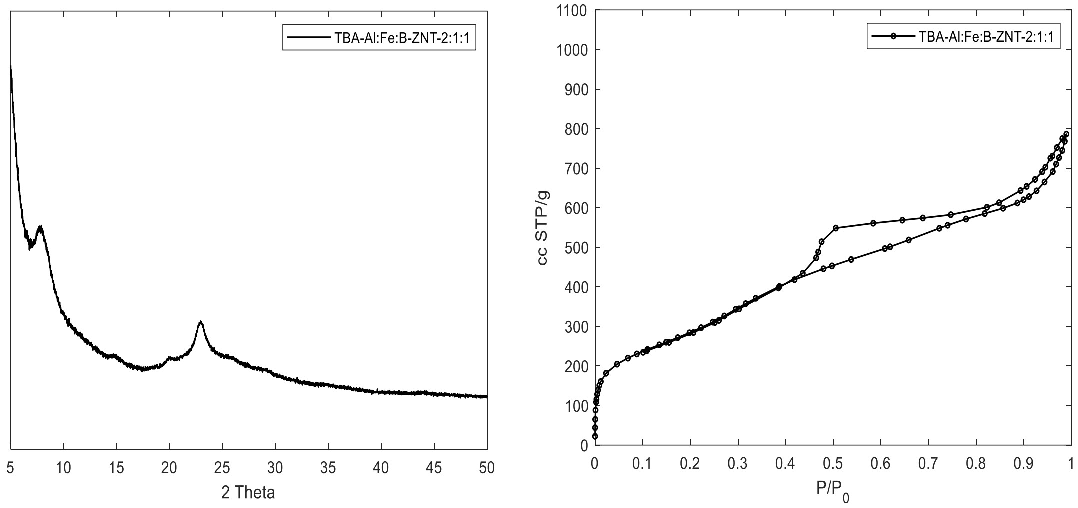

Figure S13. XRD pattern and N_2 physisorption isotherm of TBA-Al:Fe:B-ZNT-2:1:1
